# RNA sequencing and proteomics approaches reveal novel deficits in the cortex of *Mecp2*-deficient mice, a model for Rett syndrome

**DOI:** 10.1186/s13229-017-0174-4

**Published:** 2017-10-24

**Authors:** Natasha L. Pacheco, Michael R. Heaven, Leanne M. Holt, David K. Crossman, Kristin J. Boggio, Scott A. Shaffer, Daniel L. Flint, Michelle L. Olsen

**Affiliations:** 1 Department of Cell, Developmental, and Integrative Biology, University of Alabama at Birmingham, 1918 University Blvd, Birmingham, AL 35294 USA; 2 Vulcan Analytical, LLC, 1500 1st Ave. North, Birmingham, AL 35203 USA; 3 UAB Heflin Center for Genomic Science, Department of Genetics, University of Alabama at Birmingham, Kaul 424A, 1720 2nd Ave. South, Birmingham, AL 35294 USA; 4 Proteomics and Mass Spectrometry Facility, Department of Biochemistry and Molecular Pharmacology, University of Massachusetts Medical School, 222 Maple Ave., Fuller Building, Shrewsbury, MA 01545 USA; 5 Luxumbra Strategic Research, LLC, 1331 South Eads St, Arlington, VA 22202 USA; 6 School of Neuroscience, Virginia Polytechnic and State University, Life Sciences Building Room 213, 970 Washington St. SW, Blacksburg, VA 24061 USA

**Keywords:** Transcriptome, Proteome, Rett syndrome, Multi-cellular deficits

## Abstract

**Background:**

Rett syndrome (RTT) is an X-linked neurodevelopmental disorder caused by mutations in the transcriptional regulator MeCP2. Much of our understanding of MeCP2 function is derived from transcriptomic studies with the general assumption that alterations in the transcriptome correlate with proteomic changes. Advances in mass spectrometry-based proteomics have facilitated recent interest in the examination of global protein expression to better understand the biology between transcriptional and translational regulation.

**Methods:**

We therefore performed the first comprehensive transcriptome-proteome comparison in a RTT mouse model to elucidate RTT pathophysiology, identify potential therapeutic targets, and further our understanding of MeCP2 function. The whole cortex of wild-type and symptomatic RTT male littermates (*n* = 4 per genotype) were analyzed using RNA-sequencing and data-independent acquisition liquid chromatography tandem mass spectrometry. Ingenuity® Pathway Analysis was used to identify significantly affected pathways in the transcriptomic and proteomic data sets.

**Results:**

Our results indicate these two “omics” data sets supplement one another. In addition to confirming previous works regarding mRNA expression in *Mecp2*-deficient animals, the current study identified hundreds of novel protein targets. Several selected protein targets were validated by Western blot analysis. These data indicate RNA metabolism, proteostasis, monoamine metabolism, and cholesterol synthesis are disrupted in the RTT proteome. Hits common to both data sets indicate disrupted cellular metabolism, calcium signaling, protein stability, DNA binding, and cytoskeletal cell structure. Finally, in addition to confirming disrupted pathways and identifying novel hits in neuronal structure and synaptic transmission, our data indicate aberrant myelination, inflammation, and vascular disruption. Intriguingly, there is no evidence of reactive gliosis, but instead, gene, protein, and pathway analysis suggest astrocytic maturation and morphological deficits.

**Conclusions:**

This comparative omics analysis supports previous works indicating widespread CNS dysfunction and may serve as a valuable resource for those interested in cellular dysfunction in RTT.

**Electronic supplementary material:**

The online version of this article (10.1186/s13229-017-0174-4) contains supplementary material, which is available to authorized users.

## Background

Rett syndrome (RTT) is an X-linked neurodevelopmental disorder that annually affects 1:10,000–15,000 females worldwide. RTT is characterized by apparently normal development until approximately 6–18 months of age, when patients exhibit a decrease in motor and cognitive functions [1]. Additional clinical features of RTT patients include hand stereotypies, breathing abnormalities, gastrointestinal dysfunction, and seizures [1]. RTT patients require life-long, constant care limited to palliative procedures.

Approximately 95% of RTT patients have a mutation in the transcriptional regulator methyl-CpG-binding protein 2 (*MECP2*) gene [2, 3]. MeCP2 is a global transcription factor that can activate and repress transcription [4, 5], with additional roles in RNA splicing and chromatin compaction [6–9]. While MeCP2 is expressed in multiple tissues throughout the body, it is most highly expressed in the brain, specifically in the cortex and cerebellum [10, 11]. MeCP2 is most highly expressed in post-mitotic neurons compared to other CNS cell types [12, 13], with recent work indicating MeCP2 expression in other neural cell populations including astrocytes, microglia, and oligodendrocytes [14–18].

To elucidate the pathophysiology of RTT and identify potential therapeutic targets, multiple laboratories have investigated gene expression profiles in *Mecp2*-deficient mice, rat, and postmortem human tissue utilizing microarray as well as RNA sequencing (RNA-Seq) [4, 9, 19–34]. Collectively, these studies found significantly altered gene transcription, in some instances hundreds of genes, and provided valuable insight regarding additional transcriptional roles of MeCP2 for miRNA and long non-coding RNA [27–29, 35]. In addition to global gene expression studies, few groups have examined global protein expression changes in a RTT mouse [36] and zebrafish [37] model, as well as RTT patients [38, 39]. With recent advances in the sensitivity that can be obtained from mass spectrometry-based proteomics, there is an increasing need to incorporate multi-omics approaches in understanding the relationship between gene and protein expression in various disease pathologies.

Accordingly, we postulated that examination of global protein expression in RTT as well as a simultaneous comparison in data obtained between two omics platforms from the same animal model and tissue region may elucidate unknown RTT pathophysiology. In the present study, RNA-Seq, proteomics, and comparison of our data to publicly available databases of cellular brain gene expression patterns were applied to better understand RTT pathophysiology. We performed these studies in cortical tissue obtained from the Jaenisch murine model of RTT, a commonly used model that recapitulates many aspects of RTT pathophysiology, including hind limb clasping, decreased mobility, breathing abnormalities, abnormal gait, and seizures [40, 41]. Our data support recent works indicating widespread cellular dysfunction in RTT and point toward novel molecular targets in cellular metabolism, protein stability, cytoskeletal structure, and calcium signaling.

## Methods

### Animals

All experimental protocols were followed according to NIH guidelines and approval from the Animal Care and Use Committee of the University of Alabama at Birmingham. Wild-type (WT) males were bred with heterozygous *Mecp2* ^*tm1.1Jae/+*^ (Jaenisch) female mice [40]. The founding heterozygous *Mecp2* ^*tm1.1Jae/+*^ female mice and WT male mice (C57BL/6J) were obtained from the Mutant Mouse Resource and Research Centers (MMRRC). Colonies were occasionally refreshed with WT male mice from Charles River Laboratories (C57BL/6NCrl) for breeding with *Mecp2* ^*tm1.1Jae/+*^ female mice. Genotypes of the resulting offspring were validated by PCR of DNA collected from tail clips. Mutant male mice (*Mecp2* ^*Jae/y*^) along with their WT littermates were collected after postnatal day 60 (P60^+^), which represents the time point when *Mecp2*-deficient males are considered to be “symptomatic.” We define symptomatic as exhibiting hind limb clasping upon suspension from tail and decreased mobility [40], both of which were observed in all *Mecp2*-deficient males at the time of collection. For both RNA sequencing and proteomics experiments (methods outlined below), an *n* of 4 biological replicates per genotype were used, with WT littermate animals used as controls. The number of biological replicates was chosen based on a previous study examining the relationship between the number of biological replicates, RNA-sequencing depth, and statistical power [42].

### RNA and protein isolation

Animals were anesthetized under CO_2_ followed by rapid decapitation. Whole brains were dissected in ice-cold phosphate-buffered saline and then separated into the cerebellum, brain stem, midbrain, and hippocampus. One hemisphere of the whole cortex was dedicated for RNA and the other for protein isolation. RNA was isolated by placing the cortical hemisphere dedicated for RNA in 1 mL of RNAlater™ solution (Invitrogen/Thermo Fisher Scientific) and allowed to react at 4 °C for at least 1 week. RNA was collected using the Invitrogen™ Ambion™ PureLink™ RNA Mini Kit (Fisher Scientific) according to the manufacturer’s instructions, with the following modifications: (1) tissue was homogenized in 1 mL lysis buffer with β-mercaptoethanol (Sigma-Aldrich) in a dounce homogenizer 5 times and rested on ice for 5 min, followed by another 5 rounds of homogenization; (2) approximately 15 mg of the homogenate was removed and brought up to a final volume of 600 μL in lysis buffer with β-mercaptoethanol (Sigma-Aldrich), then re-homogenized as described above. RNA was eluted in 30 μL of autoclaved and filtered Mill-Q® water. Proteins were isolated from the remaining cortical hemisphere in ice-cold lysis buffer (100 mM Tris base, pH 7.5 at room temperature, 1% (*w*/*v*) SDS) with protease inhibitor cocktail and phosphatase inhibitor cocktail 3 (Sigma-Aldrich, product numbers P8340 and P0044, respectively) such that the final concentration was 40 mg/mL. Lysates were sonicated using the Model 120 Sonic Dismembrator (Fisher Scientific) for 7 s at 70% amplitude, pulse 20 s, and rest 50 s for 2 cycles. Protein lysates were centrifuged to pellet debris. The supernatant was removed into another tube and quantified using a bicinchoninic acid (BCA) assay kit (Pierce/Thermo Scientific).

### RNA sequencing

RNA samples were submitted to the Genomics Core Laboratory in the Heflin Center of Genomic Sciences at the University of Alabama at Birmingham for sample preparation and sequencing. The samples were first DNase-treated and assessed for total RNA quality using the Agilent 2100 Bioanalyzer, followed by 2 rounds of polyadenylate positive (poly A+) selection and conversion to cDNA. RNA sequencing was performed on the Illumina HiSeq 2500 using the latest versions of sequencing reagents and flow cells, providing up to 300 GB of sequence information per flow cell. TruSeq library generation kits were used according to the manufacturer’s instructions (Illumina). Library construction consisted of random fragmentation of the poly A+ mRNA, followed by cDNA production using random primers. The ends of the cDNA were repaired, A-tailed, and adaptors ligated for indexing (up to 12 different barcodes per lane) during the sequencing runs. The cDNA libraries were quantitated using qPCR in a Roche LightCycler 480 with the Kapa Biosystems kit for library quantitation (Kapa Biosystems) prior to cluster generation. Clusters were generated to yield approximately 725K–825K clusters/mm^2^. Cluster density and quality were determined during the run after the first base addition parameters were assessed. Paired-end 2 × 50 bp sequencing runs were performed to align the cDNA sequences to the reference genome mouse mm10. Approximately 15 million paired 50 bp reads were obtained per sample.

### RNA-Seq bioinformatics analysis

All RNA-Seq fastq reads (GEO Series accession number GSE96684) were processed in the Galaxy platform [43]. First, raw RNA-Seq reads were concatenated using the “Concatenate datasets tail-to-head” tool. The concatenated raw fastq reads were then trimmed using Trim Galore! (Galaxy version 0.4.2; [44]) to remove adapter sequences and low-quality base pairs with the following parameters: selected “paired-end” library; trimming reads—automatic detection; trims 1 bp off every read from its 3′ end—yes; no removal of *N* bp from 3′ end of reads 1 and 2 (respectively); and all other settings left at default. Trimmed fastq reads were then run through FastQC (Galaxy version 0.65; [45]) for additional quality control measures. Following quality control, the reads were aligned to the mouse mm10 reference genome using TopHat (Galaxy version 2.1.0; [46, 47]) using the following parameters: mean inner distance between mate pairs—175; standard deviation for distance between mate pairs—20; report discordant pair alignments—yes; and all other settings left at default. Aligned reads were assembled using Cufflinks (Galaxy version 0.0.7; [46, 48]) using the following parameters: max intron length—300,000; min isoform fraction—0.1; pre-mRNA fraction—0.15; perform quartile normalization—yes; use reference annotation—use reference annotation, with the iGenomes UCSC mm10 genome used as the reference annotation [49]; perform bias correction—yes, reference sequence data—locally cached, using reference genome—mouse mm10; use multi-read correct—yes; and job resource parameters—left at default values. Following transcript assembly and estimated fragments per kilobase of transcript per million fragments mapped (FPKM) abundances, each sample’s assembled transcript was merged using the Cuffmerge (Galaxy version 2.2.1.0; [46, 48]) tool with the following parameters: use reference annotation—yes, reference annotation—iGenomes UCSC mm10 genome [49]; use sequence data—no; minimum isoform fraction—0.05; and job resources parameters—left at default. Finally, Cuffdiff (Galaxy version 2.2.1.3; [46, 48]) was used to calculate statistical changes in gene expression using the following parameters: transcripts—output file from Cuffmerge step; omit tabular data sets—no; generate SQLite—yes; input data type—SAM/BAM; condition 1—WT TopHat accepted hits files; condition 2—*Mecp2* ^*Jae/y*^ TopHat accepted hits files; library normalization method—quartile; dispersion estimation method—per-condition; false discovery rate—0.05; minimum alignment count—100; use multi-read correct—yes; perform bias correction—yes; reference sequence data—locally cached, reference genome mouse mm10; include read group data sets—yes; include count based output files—yes; apply length correction—cufflinks effective length correction; and all other remaining parameters were left at default settings. Since poly A+ selection was utilized to generate the cDNA libraries used for RNA-Seq, any significant and differentially expressed genes that mapped to a putative non-coding gene were removed from analysis.

### Proteomics

#### In-gel tryptic digest

An amount corresponding to 40 μg of protein based on a BCA assay with BSA as a reference standard (Pierce, Rockford, IL) was processed by SDS-PAGE using a 4–20% polyacrylamide gel (Bio-Rad, Hercules, CA). The gel was run for 5 min at 120 V and stained with Coomassie Brilliant Blue R-250 protein stain comprised of 0.05% Coomassie Brilliant Blue R-250 (*w*/*v*)/50% methanol (*v*/*v*)/10% acetic acid (*v*/*v*) for 30 min at room temperature. The mobility region was excised into 1-mm cubes and destained overnight in 15% methanol (*v*/*v*)/10% glacial acetic acid (*v*/*v*). On the next day, the gel slices were destained for an additional 4 h until the stain was completely removed. The gel cubes were reduced with 10 mM dithiothreitol at 60 °C for 30 min followed by alkylation with 50 mM iodoacetamide at room temperature for 30 min. The reducing and alkylating buffers were removed, and the gel cubes were placed into acetonitrile and allowed to evaporate to dryness at room temperature. Trypsin gold (Promega, Madison, WI) in 100 mM ammonium bicarbonate was added to each sample at a 1:20 trypsin to total protein ratio and allowed to digest at 37 °C for 16 h. The digests were aliquoted into fresh tubes, and extraction buffer consisting of 50% acetonitrile (*v*/*v*)/5% formic acid (*v*/*v*) was added and left at room temperature for 2 h and then combined with each sample’s overnight trypsin digest. Samples were vacuum centrifuged to dryness, resuspended in 0.1% formic acid (*v*/*v*), and BCA assayed with undigested BSA as a reference to determine peptide total concentrations.

#### Data-independent acquisition LC-MS/MS

In each liquid chromatography mass spectrometry (LC-MS/MS) injection, 1 μg of peptides was separated on a NanoAcquity UPLC (Waters, Milford, MA). A 3-μL injection was loaded in 5% acetonitrile (*v*/*v*)/0.1% formic acid (*v*/*v*) at a 4 μL/min flowrate for 4 min onto a 100-μm I.D. fused-silica pre-column packed with 2 cm of 5 μm (200 Å) Magic C18AQ (Bruker-Michrom, Auburn, CA) and eluted using a flowrate of 300 nL/min onto a 75-μm-inner-diameter analytical column packed with 25 cm of 3 μm (100 Å) Magic C18AQ particles to a gravity-pulled tip. A linear gradient was applied to elute peptides from 100% solvent A consisting of 0.1% formic acid (*v*/*v*) to 35% solvent B comprised of acetonitrile containing 0.1% formic acid (*v*/*v*) in 90 min. Ions were introduced by positive electrospray ionization via liquid junction into a Q-Exactive hybrid mass spectrometer (Thermo, Waltham, MA) operating in data-independent acquisition (DIA) mode. A total of 6 injections were performed to analyze each sample corresponding to the precursor *m/z* ranges 501–552, 553.5–604.5, 606–657, 658.5–709.5, 711–762, and 763.5–814.5 using a modified application of the precursor acquisition independent from ion count (PAcIFIC) approach [50]. The sequential inclusion list for each precursor *m/z* range included 35 precursor *m/z* centers and stepped 1.5 *m/z* per inclusion list center to provide an overlap of 1 *m/z* between each inclusion list precursor *m/z* range assayed. Centroid MS/MS data were acquired at 17,500 FWHM resolution with an AGC target value of 2e5, a maximum IT fill time of 80 ms, an isolation width of 2.5 *m/z*, a fixed first mass of 140 *m/z*, normalized collision energy of 27, and default charge state of 2.

#### Proteomics data analysis

Protalizer version 2.1 (Vulcan Analytical, Birmingham, AL) was used to automate the analysis of DIA data and combine the results from the 6 injections per sample (ProteomeXchange Consortium/PRIDE partner repository [51, 52] data set identifier PXD006460). Peptides and source proteins were identified by an X! Tandem Sledgehammer MS/MS database search following deconvolution of MS/MS spectra as previously described by retaining fragment ions within 70% of the maximum intensity of each fragment ion when compared across sequential MS/MS scans using a 0.01 *m/z* tolerance [53]. The mouse Swiss-Prot reference proteome was used for all searches that was downloaded on March 17, 2015, and contained 6704 sequences (not including reversed sequence decoys used to determine the false discovery rate). A precursor tolerance of 2.5 *m/z* and fragment tolerance of 20 ppm were applied with a maximum of 2 missed cleavages and false protein discovery rate of 1%. Potential modifications included in each search were oxidation of M residues, pyro-glutamic acid at N-terminal E and Q residues, N-terminal acetylation, and phosphorylation at S, T, and Y sites. Carbamidomethylation of C residues was searched as a fixed modification. Peptides assigned to different proteins in separate files were assigned to a single top match determined by the protein entry most often assigned by X! Tandem. Relative quantification of peptides was performed by MS2 area-under the-curve (AUC) chromatogram intensities using a minimum of 5 and maximum of 9 b/y fragment ions in either *a*  +1 or +2 charge state with intensities at least 10% of the strongest fragment ion assigned to each peptide in MS/MS spectra. Peptides not consistently detected in each file that were acquired with the same PAcIFIC assay were extracted in the files they were not detected using normalized retention time as described elsewhere [53]. The intensities of peptide MS2 chromatograms were normalized by up to 25 peptides quantified in each sample that had the least amount of intensity variation and the most similar retention time to correct for retention time-dependent matrix effects [53]. The normalized MS2 chromatograms for each peptide were then placed in a relative scale with 1 being the smallest amount detected.

To calculate protein-level relative abundance across the *Mecp2* ^*Jae/y*^ and WT mice, each peptide assigned to a protein that was detected in every file compared was used for quantification. Proteins lacking at least one peptide detected in every sample were quantified with all the peptides that had MS2 chromatograms detected in any file. The median relative abundance of multiple peptides used for protein-level quantification was applied to determine the overall relative protein abundance of the *Mecp2* ^*Jae/y*^ mice versus WT. Statistical significance of protein-level differences was determined using an unpaired *t*-test for all the peptides used to quantify each protein using a *p*-value cutoff of *p* < 0.1 as previously applied [54].

### Western blotting

Lysates were boiled in 2× sample loading buffer (100 mM Tris base, pH 6.8, 4% SDS in Laemmli-sodium dodecyl sulfate, 600 mM β-mercaptoethanol, 200 mM dithiothreitol (DTT), and 20% glycerol) in a 1:1 ratio for 15 min at 60 °C. A final concentration of 10 μg per sample was loaded into a 4–20% gradient pre-cast mini-PROTEAN® TGX™ gel (Bio-Rad) and ran at 200 V in 1× running buffer (24.76 mM Tris base, 190 mM glycine, 0.1% SDS).

For blots tested for RNPEP, QDPR, and CIRBP protein expression, gels were transferred to a nitrocellulose membrane using the Trans-blot turbo system (Bio-Rad), mixed molecular weight protocol (2.5 A, 25 V for 7 min), followed by blocking with LI-COR® blocking buffer at a 1:1 ratio with TBS. The blots were incubated with primary polyclonal chicken anti-GAPDH (EMD Millipore) at 1:2500 for 15 min at room temperature, while the remaining primary antibodies were incubated overnight at 4 °C: polyclonal rabbit anti-RNPEP (Proteintech) at 1:1000, polyclonal rabbit anti-QDPR (Proteintech) at 1:1000, and polyclonal rabbit anti-CIRBP (Proteintech). All secondary antibodies were either goat anti-rabbit or goat anti-chicken (LI-COR®), all incubated at 1:10,000 for 1 h at room temperature. Imaging was performed on a LI-COR® Odyssey machine with 1 or 1.5 intensity on both the 680 and 800 channels. All rabbit antibodies were imaged in the 680 channels and chicken in 800.

For blots tested for MeCP2, NEFM, mGluR3, and HSPH1 protein expression, the gels were transferred to an Immobilon-P PVDF membrane (EMD Millipore) for 1 h at 100 V in 1× transfer buffer (250.7 mM Tris base, 190 mM glycine, 20% methanol) with an ice pack at room temperature, followed by blocking with 10% non-fat dry milk/0.1% Tween-20/TBS (10% milk-TBST) for 1 h at room temperature. The blots were initially incubated with primary antibody chicken anti-GAPDH at 1:2000 (EMD Millipore) for 1 h at room temperature, washed 3 times in 10% milk-TBST, and incubated for 1 h at room temperature with secondary antibody goat anti-chicken at 1:2000 (Santa Cruz Biotechnology). Blots were then washed 3 times with 10% milk-TBST and imaged with Classico chemiluminescent reagent (EMD Millipore) using an autoradiography film developing system (Denville Scientific). The blots were then individually probed with the following antibodies and conditions: mouse anti-MeCP2 at 1:2000 (Sigma) for 2 h at room temperature, rabbit anti-NEFM at 1:1000 (Proteintech) for 2 h at room temperature, rabbit anti-mGluR3 at 1:500 (Alomone labs) overnight at 4 °C, and rabbit anti-HSPH1 at 1:150 (Novus Biologicals) overnight at 4 °C. The respective secondary antibodies were incubated with blots at 1:2000 for 1 h at room temperature: goat anti-mouse (Santa Cruz) and goat anti-rabbit (Santa Cruz). Protein quantification for all blots was performed using Image Studio Lite (LI-COR®) and Origin2015 (OriginLab). Relative protein amounts per antibody tested were normalized to GAPDH expression in the same lane.

### Pathway analysis

Ingenuity® Pathway Analysis (IPA®; Qiagen) was used to identify significant biological pathways in both RNA-Seq and proteomics data sets. A list of detected genes and detected proteins (including post-translational modifications) was used as the data input for both individual and comparison pathway analyses, using a *q* < 0.05 cutoff for the gene pathway and *p* < 0.1 cutoff for the protein pathway analyses [54] such that only significant genes/proteins were considered for significant pathways. The “User dataset” option was chosen to use each individual detected gene/protein data set as the “reference set” for which to generate significant pathways. Pathways from the “diseases and biological functions” category were used for comparison analyses. Fisher’s *t*-test of *p* < 0.05 (or −Log_10_ *p*-value > 1.3) was used to determine statistical significance of a pathway.

## Results

### Global gene expression in symptomatic *Mecp2*^*Jae/y*^ whole cortex

**Fig. 1:**
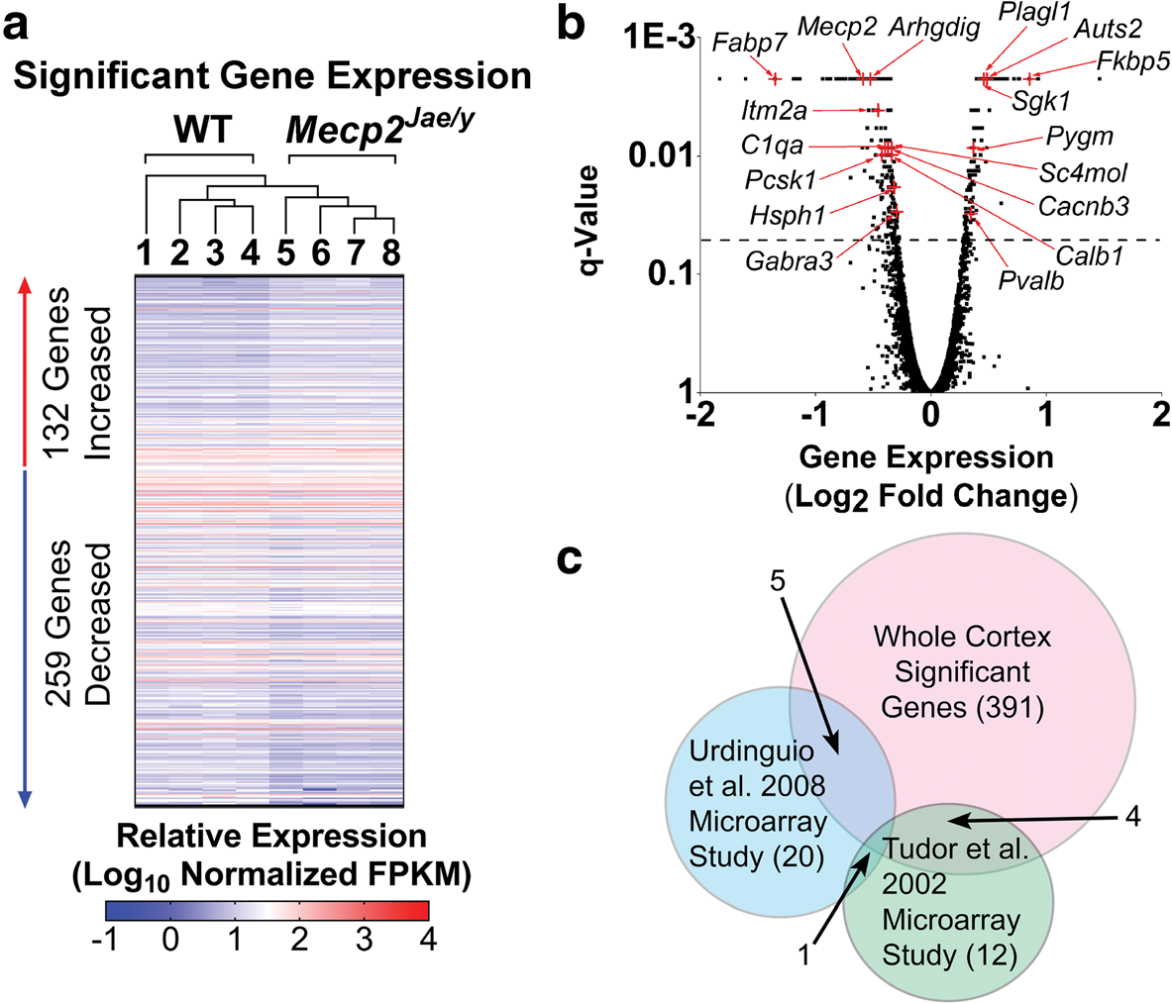
Transcriptome-wide expression in *Mecp2* ^*Jae/y*^ cortex. **a**. Heat map of 391 significant, differentially expressed (DE) genes. Each genotype has 4 biological replicates, where each column represents 1 biological replicate and each row represents the Log_10_-transformed FPKM of a significant DE gene. Biological replicates are listed in the order of how they cluster, which is indicated by the cluster dendrogram above the heat map. Genes with a false discovery rate (*q*-value or FDR) of < 0.05 were considered to be significantly, differentially expressed. **b**. Volcano plot of all the detected genes’ expression (Log_2_ fold change) in the *Mecp2* ^*Jae/y*^ whole cortex transcriptome. Significant DE genes previously identified as RTT hits are highlighted in red crosses and arrows. Due to space constraints, additional genes identified in supplemental material from Chahrour et al. [4] and Veeraragavan et al. [34] were not highlighted in this volcano plot; for information on these genes, see Table 1. Dotted line indicates a *q*-value of 0.05, where anything above the line indicates a significant DE gene. **c**. Venn diagram comparing our transcriptome data to previously published microarray studies (Urdinguio et al. [24] and Tudor et al. [20]) on *Mecp2* ^*Jae/y*^ mouse cortex. Note that in the Urdinguio study, the fold change expression was not differentiated between cortex, midbrain, and cerebellum due to their finding that there were no differences in gene expression between the 3 brain regions [24]; rather, fold change values represent combined tissue expression. Six genes were shared between the *Mecp2* ^*Jae/y*^ transcriptome data and the Urdinguio et al. study, while 5 genes from the Tudor et al. study were shared in common with the transcriptome data. One of the targets (*Fabp7*) from the Tudor et al. study was also overlapped with the Urdinguio et al. study

We began our studies by performing RNA-Seq in whole cortical tissue obtained from the *Mecp2* ^*Jae/y*^ murine model of RTT. Previous studies have analyzed the transcriptome of RTT mice; however, analysis performed in the same affected brain region in the same animal model allowed us to make general comparisons across RNA-Seq and proteomics platforms. While each data set offers unique insight into the disease, the comparison of the two allows us to make general assumptions that (1) differentially expressed “hits” common to both platforms possibly share a transcriptional mechanism of dysregulation and (2) proteins differentially expressed without a similar change in gene expression may indicate differences in either protein stability or posttranscriptional regulation. We chose to analyze whole cortex (WCX) in a *Mecp2* ^*Jae/y*^ murine model of RTT because MeCP2 protein is highly expressed in WCX [10, 11] and has pathological characteristics of RTT [26]. RNA sequencing (RNA-Seq) was used to quantify gene expression of 15 million paired 50 bp reads in WT and symptomatic *Mecp2* ^*Jae/y*^ mice (as described in the “Methods” section). We identified 391 significant, differentially expressed (DE) genes (Fig. 1a, Additional file 1). Of these 391 genes, 132 genes had increased expression and 259 had decreased expression. To assess the consistency of our data with previously published microarray and RNA-Seq studies of RTT, we compared our findings to transcriptome-based data on different species and brain regions [4, 19–26, 33, 34, 55, 56]. In our data, 35 genes were identified as known RTT differentially expressed genes and are represented in Fig. 1b and Table 1. Of the 35 genes, 18 were identified in supplemental material from [4] and [34] (Table 1). Additionally, within the group of 35 identified RTT hits, we found that 66% of the DE genes had decreased expression, while 34% had increased expression (Fig. 1b, Table 1). These observations are consistent with previous studies with the exception of one gene *Cacnb3*. In our data set, *Cacnb3* is significantly decreased; however, Tudor et al. found that this same gene was upregulated [20].

**Table 1:** Significant DE genes overlapping with previously identified RTT hits

Gene targets: this study	UniProt: function keywords	Fold change	RTT gene hits: direction of expression (with references)
Aacs*	• Molecular function: ligase • Biological process: fatty acid metabolism; lipid metabolism • Ligand: ATP-binding; nucleotide-binding	− 0.79	Decrease^4^
Aldh1a1*	• Molecular function: oxidoreductase • Ligand: NAD	− 0.67	Decrease^4^
***Arhgdig***	• **Molecular function: GTPase activation**	**− 0.69**	**Decrease** ^**2**^
*Auts2*	N/A	1.40	Increase^7^
*C1qa*	• Biological process: complement pathways; immunity; innate immunity	− 0.76	Decrease^7^
***Cacnb3***	• **Molecular function: calcium channel; ion channel; voltage-gated channel** • **Biological process: calcium transport; ion transport; transport** • **Ligand: calcium**	**− 0.79**	**Increase** ^**2**^
***Calb1***	• **Ligand: calcium; metal-binding; vitamin D**	**− 0.79**	**Decrease** ^**5**^
Calr*	• Molecular function: chaperone • Ligand: calcium; lectin; metal-binding	− 0.80	Decrease^8^
Dgkg*	• Molecular function: kinase; transferase • Ligand: ATP-binding; calcium; metal-binding; nucleotide-binding; zinc	1.42	Increase^4, 8^
Ephx2*	• Molecular function: hydrolase • Biological process: aromatic hydrocarbons catabolism; detoxification; lipid metabolism • Ligand: magnesium; metal-binding	− 0.71	Decrease^4^
***Fabp7***	• **Biological process: transport** • **Ligand: lipid-binding**	**− 0.39**	**Decrease** ^**2,5**^
***Fkbp5***	• **Molecular function: chaperone; isomerase; rotamase**	**1.81**	**Increase** ^**3,4,5,7,8**^
*Gabra3*	• Molecular function: chloride channel; ion channel; ligand-gated ion channel; receptor • Biological process: ion transport; transport • Ligand: chloride	− 0.81	Decrease^6^
Gfra1*	• Molecular function: receptor	1.26	Increase^4, 8^
Grm3*	• Molecular function: G-protein coupled receptor; receptor; transducer	− 0.63	Decrease^4^
Hpcal4*	• Ligand: calcium; metal-binding	− 0.69	Decrease^4, 8^
*Hsph1*	• Biological process: stress response • Ligand: ATP-binding; nucleotide-binding	− 0.80	Decrease^3^
***Itm2a***	**N/A**	**− 0.73**	**Decrease** ^**5**^
*Mecp2*	• Molecular function: DNA-binding; repressor • Biological process: transcription; transcription regulation	− 0.66	Decrease^1, 4^
***Msmo1*** **/** ***Sc4mol***	• **Molecular function: oxidoreductase** • **Biological process: lipid biosynthesis; lipid metabolism; steroid biosynthesis; steroid metabolism; steroid biosynthesis; sterol metabolism** • **Ligand: iron; NAD**	**− 0.77**	**Decrease** ^**5**^
Myo1b*	• Molecular function: actin-binding; calmodulin-binding; motor protein; myosin • Ligand: ATP-binding; nucleotide-binding	1.32	Increase^4, 8^
*Pcsk1*	• Molecular function: hydrolase; protease; serine protease • Ligand: calcium	− 0.74	Decrease^6^
Pdia4*	• Molecular function: isomerase	− 0.71	Decrease^4, 8^
***Plagl1***	• **Ligand: metal-binding; zinc**	**1.37**	**Increase** ^**5**^
Prkcg*	• Molecular function: kinase; serine/threonine-protein kinase; transferase • Biological process: biological rhythms • Ligand: ATP-binding; calcium; metal-binding; nucleotide-binding; zinc	− 0.82	Decrease^8^
***Pvalb***	• **Molecular function: muscle protein** • **Ligand: calcium; metal-binding**	**1.27**	**Increase** ^**2**^
*Pygm*	• Molecular function: glycosyltransferase; transferase • Biological process: carbohydrate metabolism; glycogen metabolism • Ligand: nucleotide-binding; pyridoxal phosphate	1.29	Increase^6^
Qdpr*	• Molecular function: oxidoreductase • Biological process: tetrahydrobiopterin biosynthesis • Ligand: NADP	− 0.74	Decrease^4^
Rnpep*	• Molecular function: aminopeptidase; hydrolase; metalloprotease; protease • Ligand: metal-binding; zinc	− 0.78	Decrease^4^
***Sgk1***	• **Molecular function: kinase; serine/threonine-protein kinase; transferase** • **Biological process: apoptosis; stress response** • **Ligand: ATP-binding; nucleotide-binding**	**1.37**	**Increase** ^**2,3**^
Slc24a4*	• Biological process: antiport; calcium transport; ion transport; olfaction; potassium transport; sensory transduction; sodium transport; symport; transport • Ligand: calcium; potassium; sodium	1.58	Increase^4^
Slc9a3r1*	• Biological process: Wnt signaling pathway	− 0.74	Decrease^8^
Sun2*	• Biological process: meiosis	1.42	Increase^8^
Ugp2*	• Molecular function: nucleotidyltransferase; transferase • Ligand: magnesium; metal-binding	− 0.73	Decrease^4^
Zmat4*	• Molecular function: DNA-binding • Ligand: metal-binding; zinc	1.5	Increase^8^

First column represents the significant DE gene identified in this study, along with the UniProt function keyword(s) for each gene (second column) and the Log_2_ fold change expression of the gene in our data set (third column). DE genes with an asterisk (*) represent genes identified in supplemental material from Chahrour et al. [4] and Veeraragavan et al. [34]. All function keywords were found from the UniProtKB/Swiss-Prot database [131] per respective DE gene. DE genes without a published function keyword in the UniProtKB/Swiss-Prot database are represented as “N/A.” The last column represents the previously identified RTT hits’ fold change expression direction, with each superscript representing that gene’s respective references. Superscript references are as follows: (1) Amir et al. [2], (2) Tudor et al. [20], (3) Nuber et al. [21], (4) Chahrour et al. [4], (5) Urdinguio et al. [24], (6) Ben-Shachar et al. [25], (7) Lin et al. [33], and (8) Veeraragavan et al. [34]. Rows in bold font represent previously identified RTT hits that were found to be specifically DE in the cortex

We then focused on comparisons of our RNA-Seq data to microarray studies also performed in *Mecp2*-deficient cortex [20, 24]. We specifically focused on these two studies for a number of reasons. First, studies have shown brain tissue region and cell type-specific gene and protein expression differences [30, 57–60]. While we do not negate the importance of other transcriptomic studies performed in the RTT field, we chose to compare our data set to studies performed in the same MeCP2 animal model, tissue region, and time point. A RNA-Seq study by Li et al. meets this criteria [9]; however, this study only reported upregulated genes and utilized different bioinformatics approaches. We found 175 upregulated genes in the Li et al. study were also present in our data set. Of these, 84/175 genes were also significantly upregulated in our data set (*p* < 0.05). We found 5 out of the 12 genes identified in the Tudor et al. microarray study and 6 out of the 20 genes identified in the Urdinguio et al. study were also significant DE genes in our data set (Fig. 1c). A common statistical significance threshold in RNA-sequencing studies is a *q*-value less than 0.05, which is meant to reduce the number of false positives observed, but may also underestimate biologically relevant changes. Reducing the significance threshold stringency to *p* < 0.05 in our data set, we identified an additional 2 genes from the Tudor et al. (7/12) and 2 genes from the Urdinguio et al. (8/20) study. Our RNA-Seq data correlates better with the individual microarray studies than these two studies correlate to each other, which is notable given strain differences between each of the individual studies, and that the Urdinguio et al. study utilized tissue pooled from three brain regions from the *Mecp2* ^*tm1.1Bird/y*^ mouse model [24]. These genes and their putative functions as well as direction of expression levels for each hit are shown in Table 1. We identified an additional 19 gene targets previously found to be RTT gene hits in our data set that had a *p*-value of less than 0.05, but did not meet the false discovery rate, or FDR/*q*-value, cutoff of 0.05 (Additional file 2). Collectively, this suggests that our data is in concordance with previous literature on *Mecp2*-deficient mice.

In addition to known RTT hits, we also found a number of significant DE genes that have been previously identified as autism spectrum disorder (ASD) candidate genes (*q* < 0.05). For example, mutations in *Auts2* [61, 62], *Shank1–3* [63], and *Foxp1* [64, 65] have been found in various ASD patient cases. In our data set, *Auts2* and *Foxp1* are significantly increased by 0.49 and 0.29 fold, respectively, while *Shank1–3* are all decreased (*Shank1*: − 0.53 fold, *Shank2*: − 0.34 fold, *Shank3*: − 0.37 fold) (Table 1, Additional file 1). The gene expression changes observed in our data are robust, as many were identified across multiple models, different brain regions, and numerous species. Importantly, this also provides validation in utilizing this data set as an appropriate resource for comparing RNA-protein expression similarities in the RTT model.

Recent work demonstrated that MeCP2 selectively represses gene expression of long genes [30, 32]. These two studies also showed long genes are enriched in neurons and hypothesized that neuronal dysfunction in RTT could be in part due to repression of long gene expression [30, 32]. Recent studies have also indicated that disruption of long genes could also contribute to ASD pathogenesis [66–68]. For example, one study has shown that a heterozygous knockout of the autism candidate gene *Chd8* led to an increase in dysregulated long gene expression [66], while another study found many ASD candidate genes are long genes [67]. Interestingly, recent studies have shown that topoisomerase inhibitors can regulate the expression of long genes in different ASD models [67, 68], suggesting that topoisomerase inhibitors could serve as a potential therapeutic for autism. Collectively, these studies highlight the need to understand the relationship between long gene expression and ASD and other ASD-related disorders such as RTT.

**Fig. 2:**
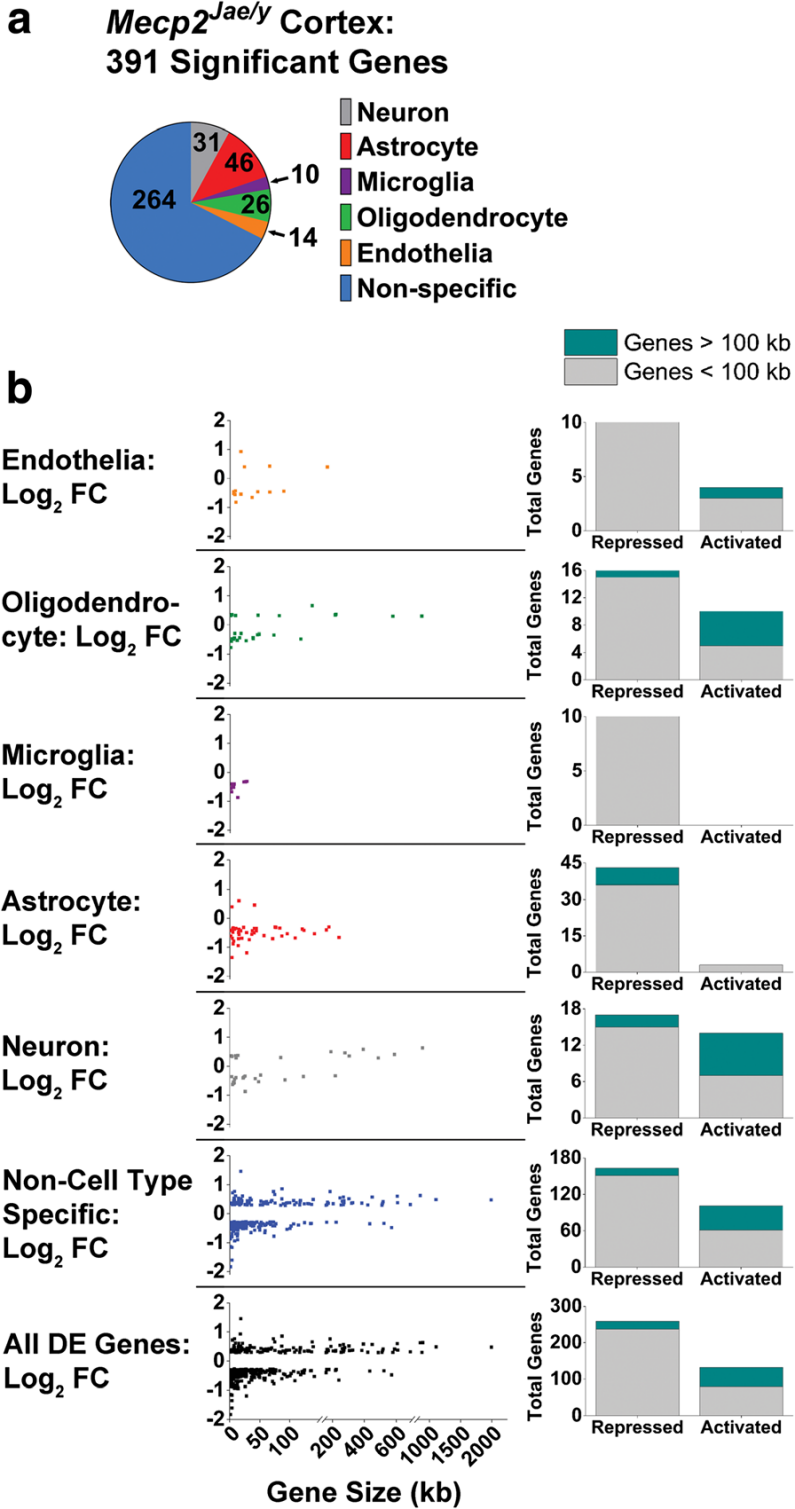
Cell type-specific gene expression correlation. **a**. *Mecp2* ^*Jae/y*^ cortex significant DE genes compared to top 500 CNS cell type-specific genes based on the Zhang et al. study [69], which is also provided as a public database. Each pie slice lists the number of DE genes associated with each CNS cell type. **b**. Fold change expression and gene size correlation in the *Mecp2* ^*Jae/y*^ transcriptome. Significant DE genes were plotted by Log_2_ fold change (FC) expression (*y*-axis) and gene size (*x*-axis; in units of kilobase (kb)) according to their respective CNS cell type distribution (based on part A). Expression and gene size correlations were also examined as a whole (bottom scatter plot in black). This relationship is also represented to the right of each scatter plot based on the number of short (defined as being less than 100 kb; gray) and long (defined as being greater than 100 kb; turquoise) genes that are either repressed (i.e., decreased expression) or activated (i.e., increased expression) in the *Mecp2* ^*Jae/y*^ cortex

Given that MeCP2 is expressed in all CNS cell types, we extended earlier studies exploring the relationship between gene size and fold change expression within cell type-specific DE genes in our data. Accordingly, we next evaluated if the 391 identified significant DE genes in symptomatic *Mecp2* ^*Jae/y*^ mice were enriched in a specific CNS cell type using a publicly available database of purified cortical CNS cell types [69]. The criteria for this comparison were as follows: (1) a gene which demonstrated a 3-fold or greater enrichment in 1 cell relative to all others was considered cell type enriched and (2) all other genes that varied less than 3-fold in any one cell type were considered to be non-specific. Using these criteria, 68% of the DE genes were not enriched in any cell type (Fig. 2a, Additional file 3). The other 32% of DE genes segregated into one enriched cell population. Of the DE genes associated with a specific cell type, 31 were enriched in neurons, 46 in astrocytes, 10 in microglia, 26 in oligodendrocytes (includes oligodendrocyte precursor cells, newly formed oligodendrocytes, and myelinating oligodendrocytes), and 14 in endothelial cells (Fig. 2a, Additional file 3). Additional comparisons to a second CNS cell type-specific database [60] further confirmed gene expression is disrupted across multiple cell populations in the symptomatic RTT brain (Additional file 4).

Based on the criteria outlined above, we cannot rule out the possibility that a gene we termed “not enriched in any cell type” is dysregulated in neurons. For example, the gene *Camk1* has strong associations with neuronal transcription and synaptic activity. However, based on our and the Zhang et al. criteria [69], *Camk1* is categorized as a “non-cell type enriched” gene. The non/cell type-specific hits may be a direct result of MeCP2 deficiency in neurons, MeCP2 deficiency in other CNS cell populations, or alternatively are downstream of MeCP2 binding in one or multiple CNS populations. We also cannot exclude the possibility that alterations observed in gene and protein expression in non-neuronal cell populations are due to the disease severity at the time point these experiments were performed. However, when we did a similar gene cell type-specific comparison between Chahrour et al.'s study and Zhang et al.'s database, we also observed a similar cell type-enriched distribution as the data presented in our study (data not shown), decreasing the possibility that our observed expression changes are due to disease severity.

We defined short genes as being less than 100 kb and long genes as larger than 100 kb based on prior studies [32] and calculated gene length based on the chromosomal location provided by the Cuffdiff differential gene expression output file. We found 81% of the total list of significant DE genes was short in our data (Fig. 2b, Additional file 5). Of these short genes, 237 had decreased and 79 had increased gene expression. Similar to previous work [30, 32], we found 71% (53/75) of the long genes exhibit increased gene expression (Fig. 2b, Additional file 5). Although the majority of genes in our data set are short and repressed, the neuronal-enriched DE genes in our data support previous studies showing a bias for over-expressed long genes in neuronal populations [30] (Fig. 2b, Additional file 5). Additionally, increased long gene expression was observed in oligodendrocyte-enriched and non-cell type-specific DE genes (Fig. 2b, Additional file 5). In contrast, the majority of astrocyte-enriched DE genes showed decreased gene expression, in which 92% (36/39) of short genes and all 7 long genes exhibited decreased gene expression (Fig. 2b, Additional file 5). Similar trends were observed in endothelial-enriched DE genes, where 71% (10/14) of the genes were short and decreased (Fig. 2b, Additional file 5). Furthermore, within the microglial-enriched DE gene group, all 10 genes are short and have decreased gene expression (Fig. 2b, Additional file 5). Together, these data provide an additional dimension of cell type-specific gene expression changes in *Mecp2* ^*Jae/y*^ animals.

### Protein expression in symptomatic *Mecp2*^*Jae/y*^ whole cortex

**Fig. 3:**
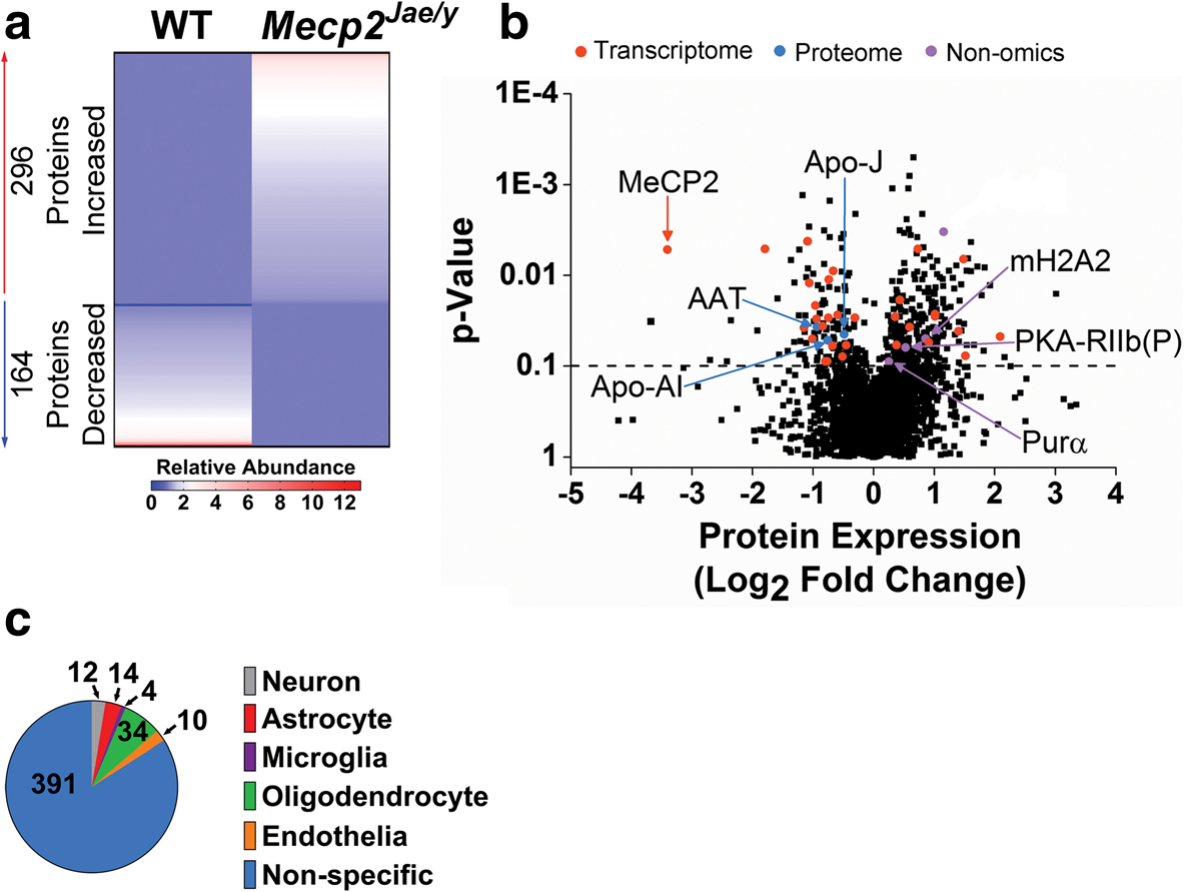
Proteome-wide expression in *Mecp2* ^*Jae/y*^ cortex. **a**. Heat map of 460 significant, abundantly expressed proteins. Each column represents pooled biological replicates per genotype (*n* = 4), and each row represents the relative abundance fold change of an individual protein (with or without a PTM). Proteins with a *p*-value of < 0.1 were considered differentially abundant. **b**. Volcano plot of all the detected proteins’ expression (Log_2_ fold change) in the *Mecp2* ^*Jae/y*^ whole cortex proteome. Dotted line indicates a *p*-value of 0.1, where anything above the line indicates a significant protein. Previously identified RTT hits are highlighted in red, blue, or purple filled circles. Red circles denote that the significant protein was identified from a transcriptome-based gene expression study, blue circles denote identification from a proteomics-based study, and purple circles denote identification from a non-omics-based study. Due to space constraints, only the selected RTT protein hits identified from proteomics and non-omics-based studies were highlighted in this volcano plot. For a comprehensive list of all the identified RTT protein hits, refer to Additional file 8. **c**. Pie chart of significant proteins compared to top 500 cell type-specific genes based on the Zhang et al. study [69]. All significant proteins were included in the analysis. Each pie slice lists the number of significant proteins associated with each CNS cell type

To gain a more complete view of cellular and molecular dysfunction in RTT, we examined global protein abundance changes in symptomatic *Mecp2* ^*Jae/y*^ whole cortex lysates using a DIA LC-MS/MS proteomics approach (see the “Methods” section; [50, 53]). We identified 465 significant protein changes out of 4789 total quantified proteins. Of the 465 significant proteins, 299 proteins had increased fold changes in RTT mice while 166 had decreased fold changes compared to WT (Fig. 3a, Additional file 6). To demonstrate the robustness and sensitivity of the expression changes observed in our proteomics data set, we performed Western blot analysis on 3 top-ranking proteins based on large fold change differences (fold change greater than ± 3; MeCP2, CIRBP, RNPEP) along with 4 proteins with smaller fold change differences (fold change less than ± 3; HSPH1, NEFM, QDPR, mGluR3) (Additional files 6 and 7). As expected, the MeCP2 protein was one of the most highly decreased and significant proteins in the data set and was also significantly decreased by Western blot analysis (Additional files 6 and 7). Western blot analysis also confirmed that the remaining 6 targets were significantly different in the *Mecp2*-deficient cortex (Additional file 7). Additionally, representative mass spectrometry chromatograms confirmed qualitative changes in peptides detected in WT versus *Mecp2*-deficient cortical lysates (Additional file 7). Collectively, these results lend support to the robustness and sensitivity of our proteomics data set.

We next examined whether any of the significant proteins were previously identified RTT hits using the same criteria as outlined in the RNA-Seq analysis section. Of the 465 significant proteins, 37 were identified as previously published RTT targets, with the majority identified from previous transcriptome studies (Fig. 3b, Additional file 8). Three of the 37 hits (clusterin, Apo-J/*Clu*; alpha-1-antitrypsin 1–1, AAT/*Serpina1a*; and apolipoprotein A-I, Apo-AI/*Apoa1*; Fig. 3b, Additional file 8) were identified from the limited proteomics studies published in RTT [36–39] and have decreased expression. Two significantly increased proteins in our data set (transcriptional activator protein Pur-alpha, Purα/*Pura*, ↑1.19 fold; and core histone macro-H2A.2, mH2A2/*H2afy2*, ↑1.43 fold; Fig. 3b, Additional file 8) were previously identified as MeCP2 interacting proteins [4] as was FK506-binding protein 51 (FKBP5; Additional file 8) [21]*.*

We identified several groups of differentially abundant proteins that fall into broad categories that relate to MeCP2 function, cellular dysfunction, and disease biology (Tables 2 and 3). For instance, several proteins related to RNA metabolism, including heterogeneous nuclear riboproteins (hnRNP a0, hnRNP D0, hnRNP D-like, hnRNP H2, hnRNP L), spliceosome components (CD2BP2, CDC5L), and a transcriptional repressor (mH2A2), were observed (Table 2). Each of these proteins was significantly upregulated in RTT tissue relative to WT. In particular, cold-inducible RNA-binding protein (CIRBP), an RNA-binding protein upregulated in response to hypoxia [70], shows one of the largest fold increases in our data set (4.3-fold; Table 2). Interestingly, this protein has previously been shown to be upregulated in RTT tissue [21], although mRNA levels do not appear to be affected ([71] and our data). Similar findings were previously reported in RTT where multiple synaptic proteins showed significant changes in protein expression while no observed changes were found at the transcript level [72]. These data suggest examining global protein changes in RTT provide supplementary information to transcriptomic studies.

**Table 2:** Differentially abundant proteins associated with RNA metabolism and proteostasis

Gene name	Protein description	FC	*p-*value	General putative function
**RNA metabolism**				
***H2afy2***	**Core histone macro-H2A.2 (mH2A2)**	**1.8**	**0.050**	**Gene repression**
*Cdc5l*	Cell division cycle 5-like protein	2.2	0.013	mRNA splicing
*Cd2bp2*	CD2 antigen cytoplasmic tail-binding protein 2	1.8	0.072	mRNA splicing
*Hnrnpa0*	Heterogeneous nuclear ribonucleoprotein (hnRNP) A0	2.0	0.076	RNA-binding protein
*Hnrnpd*	Heterogeneous nuclear ribonucleoprotein (hnRNP) D0	1.4	0.029	RNA-binding protein
*Hnrnpdl*	Heterogeneous nuclear ribonucleoprotein (hnRNP) D-like	2.1	0.024	RNA-binding protein
*Hnrnph2*	Heterogeneous nuclear ribonucleoprotein (hnRNP) H2	1.5	0.034	RNA-binding protein
*Hnrnpl*	Heterogeneous nuclear ribonucleoprotein (hnRNP) L	1.8	0.077	RNA-binding protein
***Cirbp***	**Cold-inducible RNA-binding protein**	**4.3**	**0.047**	**RNA-binding protein**
*Ddx21*	Nucleolar RNA helicase 2	2.9	0.009	rRNA transcription, processing, and modification
*Hp1bp3*	Heterochromatin protein 1-binding protein 3	2.1	0.057	Transcription regulation
**Proteostasis**				
*Impact*	Protein IMPACT	1.8	0.002	Translational activation
*Uchl5*	Ubiquitin carboxyl-terminal hydrolase isozyme L5	1.6	0.037	Deubiquitylation
*Adrm1*	Proteasomal ubiquitin receptor ADRM1	3.0	0.007	Enhances Uchl5
*Ublcp1*	Ubiquitin-like domain-containing CTD phosphatase 1	3.5	0.068	Nuclear proteasome activity
*Cryab*	Alpha-crystallin B chain	−2.1	0.003	Chaperone-like activity
*Hspb1*	Heat shock protein beta-1 (HspB1)	−1.7	0.020	Chaperone for protein folding maintenance
Hsph1*	**Heat shock protein 105 kDa**	**−2.1**	**0.004**	**Chaperone activity regulation**
*Hsp90aa1*	Heat shock protein HSP 90-alpha (HSP90α)	−1.5	0.058	ATP-dependent chaperone
*Hsp90ab1*	Heat shock protein HSP 90-beta (HSP90β)	−1.4	0.056	ATP-dependent chaperone
*Hspa4*	Heat shock 70 kDa protein 4	−1.5	0.067	Molecular chaperone
*Ahsa1*	Activator of 90 kDa heat shock protein ATPase homolog 1	−1.3	0.059	Chaperone binding

Significant proteins (*p* < 0.1) that are associated with either RNA metabolism (top half of table) or proteostasis (bottom half of table) are listed by gene name (first column), followed by the full protein name (second column). For each protein, the fold change (“FC”) and *p*-value is provided (third and fourth columns, respectively), along with a brief description regarding the putative function of that protein (“General Putative Function”). Information regarding the putative function of each protein was obtained from the UniProtKB/Swiss-Prot database [131]. Gene names with an asterisk (“*”) indicates that the corresponding gene was also identified as significantly, differentially expressed (DE) in the transcriptome by a *q* < 0.05. Bold proteins indicate that the respective protein has been previously identified as a RTT hit and/or MeCP2 interacting protein (see text for references)

**Table 3:** Differentially abundant proteins associated with metabolism and S-adenosylmethionine-dependent methylation

Gene name	Protein description	FC	*p*-value	General putative function
**Metabolism**				
*Lss*	Lanosterol synthase	8.1	0.016	Cholesterol synthesis
Aacs*	**Acetoacetyl-CoA synthetase**	**− 1.4**	**0.059**	**Ketone body utilization**
*Acadvl*	Very long-chain specific acyl-CoA dehydrogenase, mitochondrial	1.6	0.045	Fatty acid oxidation
***Ugp2*** ^***#***^	**UTP-glucose-1-phosphate uridylyltransferase**	**− 1.7**	**0.011**	**Glycogen metabolism**
*Acot7*	Cytosolic acyl coenzyme A thioester hydrolase	− 1.3	0.087	Fatty Acyl-CoA biosynthesis
**Monoamine metabolism**				
***Qdpr*** ^***#***^	**Dihydropteridine reductase**	**− 1.7**	**0.029**	**Tetrahydrobiopterin synthesis**
Spr*	Sepiapterin reductase	− 1.5	0.083	Tetrahydrobiopterin synthesis
*Maoa*	Amine oxidase [flavin-containing] A	1.4	0.096	Degradation of monoamines
**S-adenosylmethionine-dependent methylation**				
***Gamt***	**Guanidinoacetate** ***N*** **-methyltransferase**	**− 1.7**	**0.089**	**Creatine synthesis**
*Srm*	Spermidine synthase (SPDSY)	− 1.5	0.024	Polyamine synthesis
*Mthfd1l*	Monofunctional C1-tetrahydrofolate synthase, mitochondrial	1.7	0.054	Folate synthesis

Significant proteins (*p* < 0.1) are listed by gene name (first column), followed by the full protein name (second column). For each protein, the fold change (“FC”) and *p*-value is provided (third and fourth columns, respectively), along with a brief description regarding the putative function of that protein (“General Putative Function”). Information regarding the putative function of each protein was obtained from the UniProtKB/Swiss-Prot database [131]. Gene names with an asterisk (“*”) indicates that the corresponding gene was also identified as significantly, differentially expressed (DE) in the transcriptome by a *q* < 0.05, while genes with a pound sign (“#”) indicate that gene was identified as DE in the transcriptome by a *q* < 0.01. Bold proteins indicate that the respective protein has been previously identified as a RTT hit and/or MeCP2 interacting protein

Our proteome data indicate cellular dysfunction in RTT may be facilitated by disrupted proteostasis (Table 2). We observed significantly decreased abundance of multiple heat shock proteins (HSPH1, HSP90α, HSP90β, and HSPA4), including the small heat shock proteins alpha-crystallin B chain (CRYAB) and HspB1 (Table 2). In support of these findings, a recent study has shown that microglia isolated from pre-symptomatic RTT female mice show decreased expression in heat shock genes [73]. Because of the important role that heat shock proteins play in regulating protein folding and stability, it is possible that expression changes in these heat shock proteins could be influencing the protein expression changes we observe in our proteomics data set. Furthermore, the protein IMPACT, which mediates translation in response to numerous cell stressors, was also upregulated in the proteome data set (1.8-fold; Table 2). Elevated physiological stress can also affect the protein expression levels of FKBP5 (elevated in response to glucocorticoid signaling [21]), the diacylglycerol-binding protein DGKγ, and the guanine nucleotide exchange factor RasGRF1 [74, 75], all of which are significantly increased in our data (Additional file 6).

Recent reports indicate altered metabolism in RTT. We observed significant expression of proteins involved in ketone body metabolism and utilization (AACS) as well as fatty acid oxidation (ACADVL) and glycogen metabolism (UGP2) (Table 3). Lanosterol synthase (LSS), a key enzyme in cholesterol synthesis, was elevated 8.1-fold in our data set (Table 3). Cholesterol synthesis has been shown to be disrupted in a suppressor screen in *Mecp2*-deficient mice [76]. It is conceivable that elevated LSS protein expression contributes to elevated cholesterol triglycerides and LDLs commonly observed in RTT patients [77, 78]. Our data also indicate altered monoamine metabolism as evidenced by significant downregulation of two key enzymes (QDPR, SPR) involved in the production of dopamine, norepinephrine, and serotonin (Table 3). The product of these enzymes is tetrahydrobiopterin (BH4), an essential cofactor in the production of amine neurotransmitters. Furthermore, amine oxidase A (MAOA), which catalyzes the oxidative deamination or degradation of these same neurotransmitters, is upregulated in the proteome data set (Table 3). Together, these findings provide additional support for prior studies indicating that dopamine, norepinephrine, and serotonin are downregulated in RTT mice and patients [79, 80].

Finally, we identified a number of significantly altered proteins associated with S-adenosylmethionine-dependent methylation (GAMT, SPDSY, and MTHFD1L; Table 3). This particular pathway converts methionine to S-adenosylmethionine (SAM), whereby the methyl group from SAM is transferred to different substrates such as DNA [81, 82]. This finding is of interest to RTT because MeCP2 binds methylated and un-methylated genomic regions to regulate gene transcription [4], and global MeCP2 methylation-binding patterns in RTT have been well documented [13, 31, 32, 83, 84]. An additional methyltransferase also had significant differential abundance between RTT and WT animals (PRMT5; Additional file 6). Methyl donor groups that are utilized by SAM-dependent methylation mechanisms include creatine, folate, folinic acid, and betaine, all of which have also been implicated in RTT [85]. Collectively, this suggests aberrant regulation of methyl donor group availability to properly regulate DNA methylation, and consequent downstream gene expression, in RTT.

### Significant proteins in symptomatic *Mecp2*^*Jae/y*^ whole cortex are expressed in multiple CNS cell types

To determine if any of the significantly abundant proteins identified in our proteomics data set were also enriched in a specific cell type, we compared the 465 significant proteins to the same CNS cell type database as the RNA-Seq data. Similar to our DE gene findings, most significantly altered proteins were non-cell type specific (Fig. 3c, Additional file 3). Of the cell type-specific significantly regulated proteins, 12 were neuronal-enriched, 14 astrocyte-enriched, 4 microglial-enriched, 34 oligodendrocyte-enriched, and 10 endothelial-enriched (Fig. 3c). Similar to the RNA-Seq data, the proteomics data also suggest that loss of MeCP2 has downstream effects on protein expression in different CNS cell populations.

It should be noted that in our study, we have solely examined gene/protein expression in whole cortical tissue homogenate. A number of studies have identified this “dilution effect” [30, 86, 87], where certain genes in one cellular population could be masked by the gene expression from a heterogeneous cellular population. Additionally, gene and proteins typically thought of as “cell type specific” may lose their specificity in the RTT brain. Future studies examining gene and protein expression from multiple cell types simultaneously isolated from the same RTT animal model and tissue region are needed to properly address these potential confounds. We also cannot exclude the possibility that changes in gene and protein expression could be due to differences in cell population number. Future studies utilizing quantitative stereology and multi-channel immunohistochemistry should be done to examine cell counts in WT versus *Mecp2*-deficient brain tissue.

### Transcriptome-proteome expression comparison identifies novel hits

**Fig. 4:**
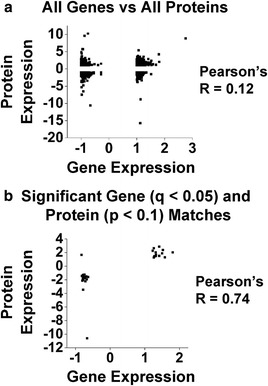
Transcriptome-proteome expression correlation in *Mecp2* ^*Jae/y*^ cortex. **a**. Overall gene-protein expression correlation. Detected genes (7026) from the RNA-Seq data set were matched against detected proteins (4789) from the proteomics data set, resulting in a total of 3780 gene-protein matches. Each individual gene-protein match is plotted by gene fold change expression (*x*-axis, *Mecp2* ^*Jae/y*^/WT) and its corresponding protein fold change expression (*y*-axis, *Mecp2* ^*Jae/y*^/WT). Pearson’s *R* reports a correlation of 0.12. **b**. Significant gene and significant protein expression correlation. Out of the 3780 detected gene-protein matches, only 35 have both a significant gene (*q* < 0.05) and corresponding significant protein (*p* < 0.1) match. Each match is plotted by gene fold change expression (*x*-axis, *Mecp2* ^*Jae/y*^/WT) and its corresponding protein fold change expression (*y*-axis, *Mecp2* ^*Jae/y*^/WT). Pearson’s *R* reports a correlation of 0.74

We next compared the proteome to the RNA-Seq data set. Previous studies making similar comparisons across species reported low correlation between these two types of data [88–91]. Our comparison across the transcriptomic and proteomics data resulted in low correlation (Pearson’s *R* of 0.12, Fig. 4a). Within the detected gene-protein matches, which we define as a detected gene having a corresponding detected protein, we identified 35 significant gene-protein matches (gene expression *q* < 0.05, protein expression *p* < 0.1) with a Pearson’s *R* of 0.74 (Fig. 4b and Table 4). When we relaxed the stringency in the DE gene list to *p* < 0.05, this value increases to 77 significant gene-protein matches with a Pearson’s *R* of 0.67 (Additional file 9).

**Table 4:** List of significant genes with a significant protein

Gene/protein	UniProt accession	Gene fold change	Protein fold change	RTT hit?	Putative function
*Mecp2*/MeCP2	Q9Z2D6	− 0.66	− 10.6	Yes^1, 2^	DNA binding protein; transcriptional regulator
*Rnpep*/RNPEP*	Q8VCT3	− 0.78	− 3.47	Yes^2^	Peptide catabolic process
*Pdia4*/ERp72*	P08003	− 0.71	− 2.22	Yes^2,3^	ER protein processing
*Hsph1*/HSPH1	Q61699	− 0.80	− 2.13	Yes^4^	Protein chaperone
*Rnpep*/RNPEP(Ac)*	Q8VCT3	− 0.78	− 2.02	Yes^2^	Peptide catabolic process
*Aldh1a1*/ALDH1A1*	P24549	− 0.67	− 1.95	Yes^2^	Retinal dehydrogenase
*Slc9a3r1*/NHERF1(P)*	P70441	− 0.74	− 1.79	Yes^3^	Scaffold protein
*Ugt8a*/UGT8	Q64676	− 0.79	− 1.76	---	Sphingolipid metabolism
*Qdpr*/QDPR (or DHPR)*	Q8BVI4	− 0.74	− 1.68	Yes^2^	Monoamine metabolism (tetrahydrobiopterin biosynthesis)
*Ugp2*/UGP2*	Q91ZJ5	− 0.73	− 1.67	Yes^2^	Glycogen synthesis
*Car2*/Ca2	P00920	− 0.72	− 1.61	---	Reversible hydration of carbon dioxide
*Grm3*/mGluR3*	Q9QYS2	− 0.63	− 1.6	Yes^2^	Protein coupled glutamate receptor
*Ephx2*/EPHX2*	P34914	− 0.71	− 1.59	Yes^2^	Cholesterol synthesis
*Hpcal4*/HPCAL4*	Q8BGZ1	− 0.69	− 1.51	Yes^2,3^	Calcium binding
*Spr*/SPR	Q64105	− 0.77	− 1.47	---	Monoamine metabolism (tetrahydrobiopterin metabolic process)
*Sash1*/SASH1	P59808	− 0.82	− 1.44	---	p38 MAPK and NIK/NF-kappaB signaling
*Calr*/CRT*	P14211	− 0.80	− 1.43	Yes^3^	ER calcium-binding protein
*Aacs*/AACS*	Q9D2R0	− 0.79	− 1.37	Yes^2^	Fatty acid and lipid metabolism
*S1pr1*/S1P1	O08530	− 0.78	− 1.28	---	Cell-cell adhesion
*Slc9a3r1*/NHERF1(Ac)*	P70441	− 0.74	− 1.24	Yes^3^	Scaffold protein
*Nova1*/NOVA1	Q9JKN6	1.26	1.28	---	RNA/mRNA-binding protein
*Slc24a4*/Nckx4*	Q8CGQ8	1.58	1.3	Yes^3^	K^+^-dependent Na^+^/Ca^2+^ exchanger
*Kcnab3*/Kvβ3.1	P97382	1.27	1.43	---	Voltage gated K+ channel
*Me3*/NADP-ME3	Q8BMF3	1.28	1.46	---	Mitochondrial NADP(+)-dependent malic enzyme
*Tfrc*/TfR	Q62351	1.32	1.48	---	Cell surface receptor required for iron uptake
*Dgkg*/DGKγ*	Q91WG7	1.42	1.51	Yes^2,3^	Lipid metabolism
*Prkcg*/PKCγ(P)*	P63318	− 0.82	1.66	Yes^3^	Protein kinase; LTP
*Rasgrf1*/RasGRF1	P27671	1.32	1.68	---	Stimulates the dissociation of GDP from RAS protein
*Zmat4*/ZMAT4*	Q8BZ94	1.50	1.88	Yes^3^	DNA/RNA and p53 binding
*Myo1b*/Myo1b*	P46735	1.32	2.02	Yes^2,3^	Actin binding; calmodulin binding
*Fkbp5*/FKBP5	Q64378	1.81	2.02	Yes^2–6^	Immunoregulation; protein trafficking
*Wipf3*/WIPF3	P0C7L0	1.23	2.26	---	Cytoskeleton organization
*Itih3*/ITI-HC3	Q61704	1.52	2.37	---	Anchor protein between hyaluronan and matrix proteins
*Gfra1*/GDNFRα1*	P97785	1.26	2.64	Yes^2,3^	RET and RAF/MAP kinase signaling cascade
*Sun2*/SUN2*	Q8BJS4	1.42	2.86	Yes^3^	Nuclear envelope protein

Significant genes were defined as having a *q* < 0.05, and significant proteins having a *p* < 0.1. Gene and protein names are provided along with the UniProt accession number, gene and protein fold change expression values (respectively), whether the gene-protein match has been previously identified as a RTT hit, and the respective putative function according to the UniProtKB/Swiss-Prot database [131]. DE gene-protein matches with an asterisk (*) represent genes/proteins identified in supplemental material from Chahrour et al. [4] and Veeraragavan et al. [34]. Gene-protein matches with “(Ac)” denote that the corresponding protein had a significant acetylation PTM, and a “(P)” denotes a significant phosphorylation PTM. For the RTT hit column, a “---” indicates the match has not been identified as a RTT hit prior to this study. Superscripts in the RTT hit column denote references for matches identified as a RTT hit and are as follows: (1) Amir et al. [2], (2) Chahrour et al. [4], (3) Veeraragavan et al. [34], (4) Nuber et al. [21], (5) Urdinguio et al. [24], and (6) Lin et al. [33]

We found 23 of the 35 significantly DE gene-protein matches were previously identified as RTT RNA regulatory targets (Table 4). The remaining 12 matches have not been described in previous RNA studies (Table 4). Within the gene-protein matches with similar regulatory patterns, *Ephx2*/EPHX2, *Dgkg*/DGKγ, *Me3*/NADP-ME3, *Qdpr*/QDPR, *Slc24a4*/SLC24A4, *Ugt8a*/UGT8, *Aacs*/AACS, *Rnpep*/RNPEP, and *Spr*/SPR are implicated in metabolic pathways (Table 4) [92–100]. We also found that *Itih3*/ITI-HC3, *Slc9a3r1*/NHERF1 (both phosphorylated and acetylated modifications), and *Rnpep*/RNPEP are associated with protein scaffolding/stability (Table 4) [99, 101–103]. Additionally, *S1pr1*/S1P1, *Calr*/CALR, *Hpcal4*/HPCAL4, and *Rasgrf1*/RasGRF1 are implicated in calcium-mediated processes (Table 4) [104–107].

### Pathway analysis in transcriptome and proteome implicate similar biological pathways

**Fig. 5:**
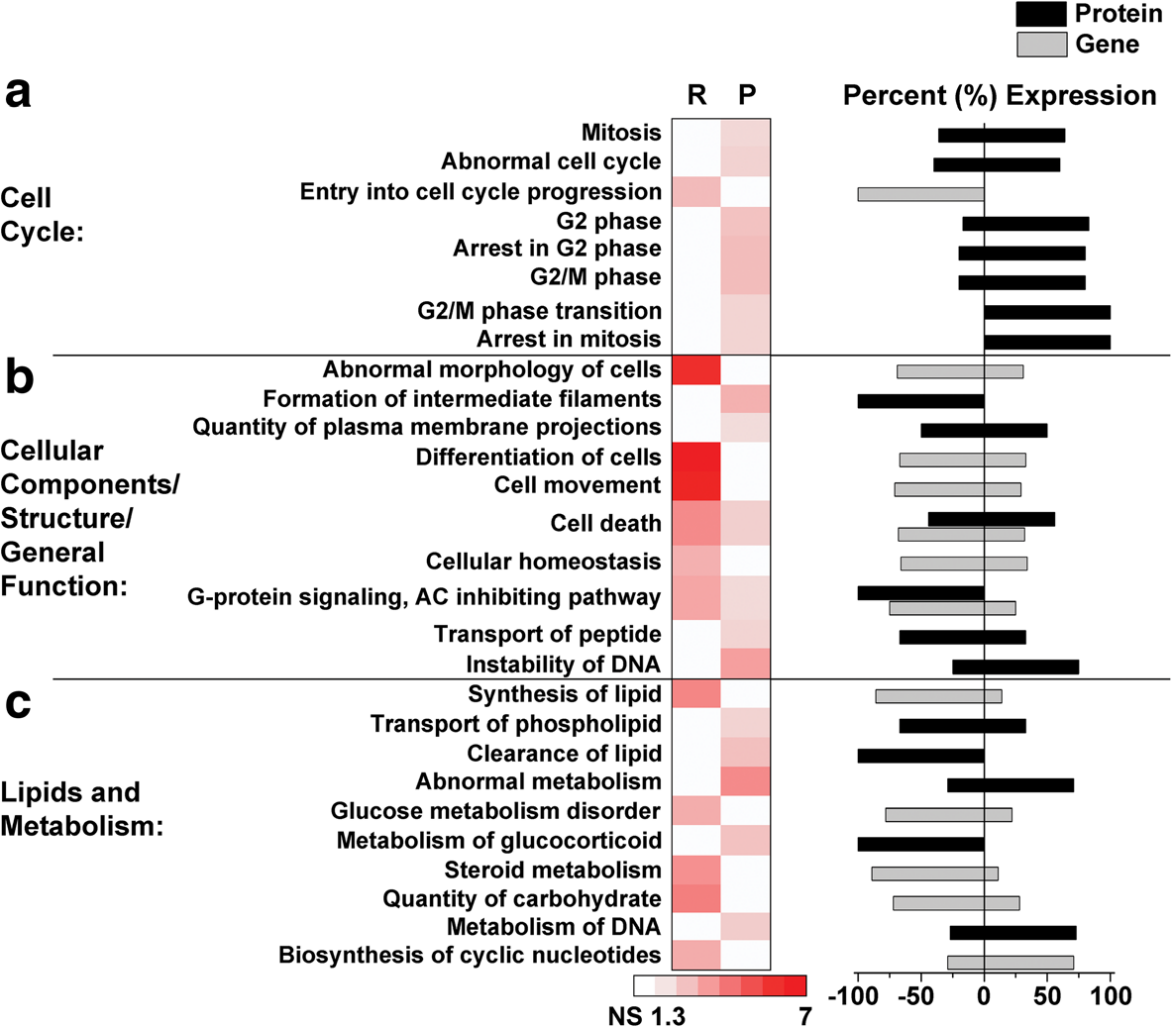
Transcriptome and proteome general cellular and molecular pathways in *Mecp2* ^*Jae/y*^ cortex. Selected biological pathways that are both shared and unique to the transcriptome (R) and proteome (P) data sets are grouped by broad categories associated with general cellular/molecular function: (**a**) cell cycle, (**b**) cellular components/structure/general function, and (**c**) lipids and metabolism. Respective pathways are plotted by −Log_10_ *p*-value, where a value of 1.3 or greater represents a *p*-value of at least *p* < 0.05; non-significant (NS) pathways are denoted in white. The direction of gene (gray bars) and/or protein (black bars) expression changes associated with each respective pathway are represented as a percent expression to the right of the heat map, where a value greater than 0 indicates increased expression and a value less than 0 indicates decreased expression. The percent expression was calculated by taking the number of genes/proteins with significant increased or decreased expression divided by the total sum of significant genes/proteins assigned to the respective pathway. Note in part B, the abbreviation “AC” in the “G-protein signaling, AC inhibiting pathway” stands for “adenylate cyclase.” For a comprehensive list of all pathways and associated significant genes/proteins identified in both the transcriptome and proteome data sets, see Additional file 10

**Fig. 6:**
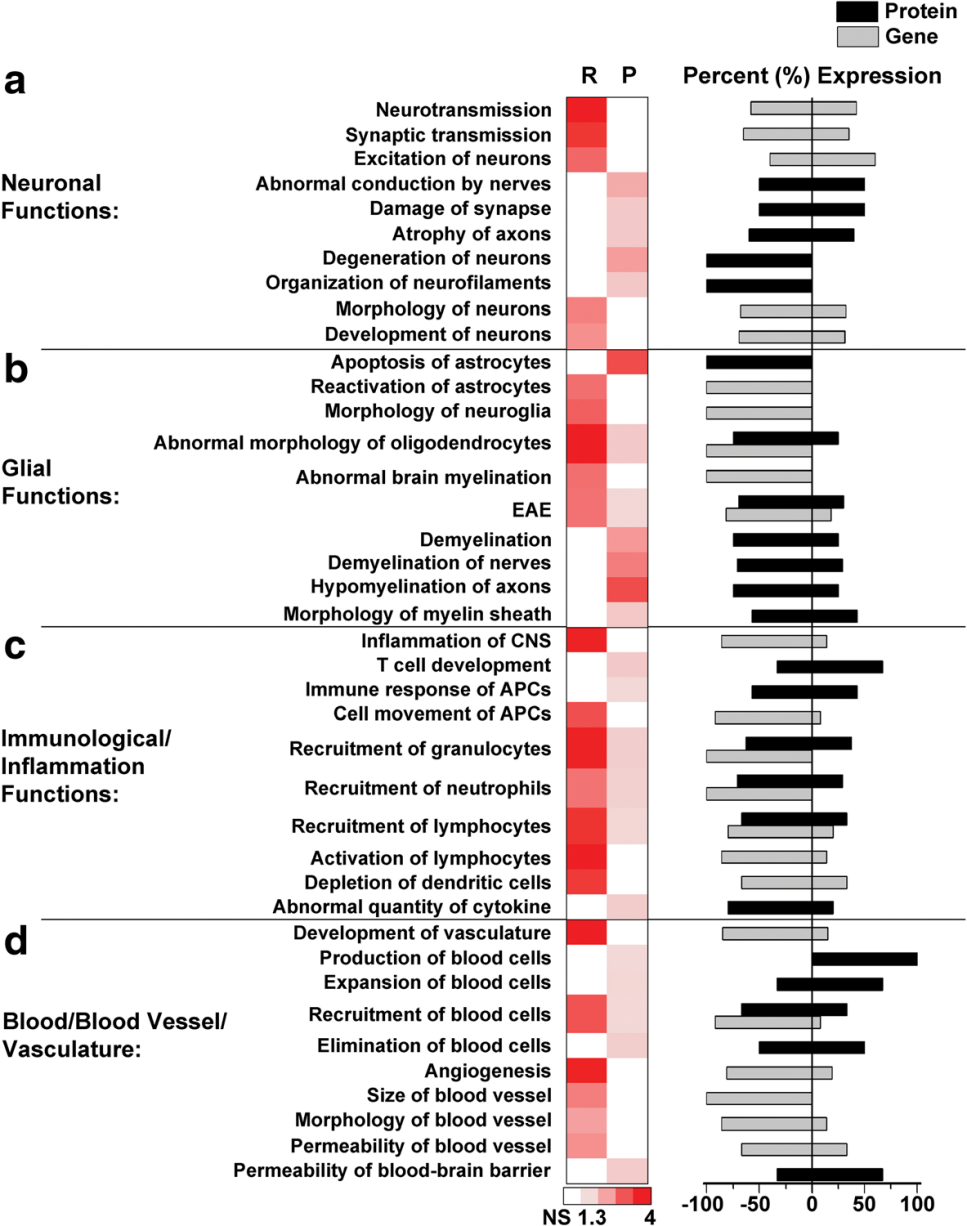
Transcriptome and proteome cell type-specific pathways in *Mecp2* ^*Jae/y*^ cortex. Selected biological pathways that are both shared and unique to the transcriptome (R) and proteome (P) data sets are grouped by broad categories associated with CNS and general cell type specific function: (**a**) neuronal functions, (**b**) glial functions, (**c**) immunological/inflammation functions, and (**d**) blood/blood vessel/vasculature. Respective pathways are plotted by −Log_10_ *p*-value, where a value of 1.3 or greater represents a *p*-value of at least *p* < 0.05; non-significant (NS) pathways are denoted in white. The direction of gene (gray bars) and/or protein (black bars) expression changes associated with each respective pathway are represented as a percent expression to the right of the heat map, where a value greater than 0 indicates increased expression and a value less than 0 indicates decreased expression. The percent expression was calculated as described in Fig. 5. Note in part B, the abbreviation “EAE” stands for “experimental autoimmune encephalomyelitis”; in part C, the abbreviation “APCs” in the pathways “Immune response of APCs” and “Cell movement of APCs” stands for “antigen presenting cells.” For a comprehensive list of all pathways and associated significant genes/proteins identified in both the transcriptome and proteome data sets, see Additional file 10

In an attempt to understand the underlying biology of disrupted genes and proteins, we utilized Ingenuity® Pathway Analysis (IPA) on the protein and RNA data sets. For a comprehensive list of all identified pathways in the individual transcriptome and proteome data sets as well as the corresponding genes and proteins in each pathway, see Additional file 10. To exemplify the diversity of identified significant pathways, we highlight functional categories that are similar or shared between the transcriptome and proteome data sets in Figs. 5 and 6. First, we identified pathways associated with general cellular and molecular dysfunction. This included categories such as cell cycle, cell components/structure/general function, and lipids and metabolism (Fig. 5a-c, Additional file 10). For the cell cycle category, 36% (4/11) of the identified pathways are associated with G_2_ and G_2_/M phase, which were found exclusively in the proteomics data set (Fig. 5a, Additional file 10). Over 80% of the proteins associated with these specific pathways had significantly increased expression, suggesting hypersensitivity and/or an accumulation of cells in the G_2_/M phases, a phenomenon previously observed in RTT primary human fibroblasts [108–110].

Within the cell components/structure/general function category, we found a number of pathways related to cell death, tissue/cellular morphology, and G-protein signaling shared between the transcriptome and proteome (Fig. 5b, Additional file 10). In the RTT brain, the primary morphologic change in humans is reduced brain size [111]. Additional morphological changes include simplified dendritic arborizations and altered spine density and morphology [41, 112]. A recent study also identified reduced astrocyte branching and overall process length [72]. Supporting this, pathway analysis identified several disrupted pathways associated with tissue and cellular morphology such as “morphology of cerebral cortex”, “abnormal morphology of cells”, “formation of intermediate filaments”, “quantity of plasma membrane projections”, and “differentiation of cells” (Fig. 5b, Additional file 10). Additionally, pathway analysis supports previous findings that metabolites including glucose [113–115], lipids/phospholipids [77, 78, 80, 115–117], TCA cycle intermediates [115], biogenic amines [79, 80], glutamate [80, 117], creatine [80, 85], and steroids such as cholesterol and glucocorticoids [76, 78, 114, 118–120] are aberrantly regulated in RTT (Fig. 5c, Additional file 10).

Recent interest in different neural cell types in RTT prompted us to examine dysfunction in cell type-specific pathways. Given the wealth of information on neuronal dysfunction in RTT, we unsurprisingly identified disrupted pathways associated with synaptic/neurotransmission, neuronal morphology, and development. Proteome pathway analysis also indicated disrupted conduction and neuronal structure/organization (Fig. 6a). These pathways and expression trends are consistent with previously published work in RTT [41, 111]. Within the glial functions group, we found the majority of pathways in both the transcriptome and proteome data sets are associated with myelination processes, along with astrocyte morphology, reactivation, and apoptosis (Fig. 6b). A high number of oligodendrocyte function-based pathways were observed in the proteome (8/9 pathways) in which 7 of the pathways have decreased protein expression (Fig. 6b). While the exact mechanisms of how oligodendrocytes contribute to RTT pathogenesis have received little attention, previous studies have implicated an abnormal oligodendrocyte/myelin involvement in the disease [17, 121–123]. Altogether, the glial pathways seem to suggest abnormal glial morphology as well as aberrant myelination functions in RTT.

We additionally identified unique pathways associated with inflammation/immunology and blood vessel/vasculature pathways as disrupted in the transcriptome and proteome data sets (Fig. 6c, d, respectively). Within the inflammation/immunology category, 90% (9/10) of the pathways had decreased gene and protein expression (Fig. 6c). Furthermore, 7 immunology/inflammation pathways were found to be shared in common with both the transcriptome and proteome data sets (Fig. 6c, Additional file 10). Indeed, our pathway analysis has identified pathways such as “T cell development”, “abnormal quantity of cytokine”, “recruitment of lymphocytes”, and “activation of lymphocytes” (Fig. 6c), in which disruptions in T cell/T lymphocyte differentiation and cytokine regulation have been documented in RTT [124, 125]. These identified immunology/inflammation pathways highlight a disrupted inflammatory component in RTT, a topic that has received considerable recent attention [124, 125].

Finally, we observed a number of pathways associated with blood cells such as “production of blood cells”, “expansion of blood cells”, “recruitment of blood cells”, and “elimination of blood cells” (Fig. 6d). This is particularly interesting as platelet defects have been observed in RTT patients with *FOXG1* mutations [126]. While few studies have examined general vasculature in RTT, defects in vascular function have been previously observed [127]. The above identified pathways suggest a dysregulated function of the vasculature, and perhaps an increased risk of blood-brain barrier breakdown (a pathway also identified in the proteomics data, Fig. 6d), in RTT symptomatic WCX.

## Discussion

To provide a more complete understanding of how MeCP2 deficiency contributes to cellular and molecular dysfunction, we performed comparative analyses between global gene and protein expression in *Mecp2*-deficient mice. Comparing the RNA-Seq data to a publicly available database of cell type-specific gene expression [69], we observed more than 30% of the identified DE genes could be ascribed to specific cell populations. We confirmed previous reports [30, 32] indicating long genes were over-expressed in the neuronal population, a pattern that was also observed in oligodendrocytic cells. In contrast, astrocytic, microglial, and endothelial genes were decreased regardless of gene size. Our proteomics data indicate hundreds of novel protein targets, including proteins involved in proteostasis, metabolism, S-adenosylmethionine-dependent methylation, and an altered stress response. A comparison between RNA-Seq and proteomics data indicates relatively low correlation between the two data sets. However, 35 significant gene-protein target hits were found common to both data sets. Together, these data may serve as a resource for those interested in cellular dysfunction in Rett syndrome.

### Why the proteome?

Much of our understanding of MeCP2 function is derived from transcriptomic studies, with the general assumption that alterations in the transcriptome correlate with proteomic changes. Challenging this central dogma, recent studies indicate that RNA-protein expression correlations are low even when comparing the same tissue samples [88–91]. There are a number of reasons given for the observed discrepancies, including differences in stability and lifetime of the two types of molecules, posttranscriptional and posttranslational modifications, and protein turnover [90, 91]. These changes are likely to be exacerbated in a disease state, particularly a disease such as RTT where data indicates RNA metabolism and proteostasis are disrupted (Table 2). Thus, the primary benefit of performing a direct comparison between transcriptomic and proteomic data is the ability to complement the knowledge gained from each of these omics technologies, providing a more holistic understanding of the interplay between gene and protein expression regulation. The benefits of such comparisons are reflected in our pathway analyses, where “neuronal transmission”, “synaptic transmission”, “excitation of neurons”, “morphology of neurons”, and “development of neurons” pathways are unique to the transcriptome data set. Supplementing these transcriptomic pathways, pathways identified using the proteome data indicated “abnormal conduction”, “damage of synapses”, “atrophy of axons”, and “organization of neurofilaments” as disrupted. These findings indicate that evaluating data obtained from multiple omics platforms provide a more complete understanding of disease biology.

While the current study has only examined transcriptomic and proteomic expression changes in symptomatic *Mecp2*-deficient male mice, we recognize that utilization of a heterozygous female *Mecp2*-deficient mouse model is more clinically relevant. To date, the majority of transcriptomic and proteomic studies have been performed in male animal models. Therefore, we chose to use symptomatic *Mecp2*-deficient male mice for comparison with existing data in the RTT field. Future studies should be directed toward understanding proteomic and transcriptomic changes occurring in the same RTT female mouse model and tissue region.

### Cell type-specific gene and protein expression

#### Neurons

MeCP2 is most highly expressed in neurons and loss of MeCP2 contributes to neuronal cell dysfunction [11, 12]. We identified 2 neuronal-enriched hits *Dgkg*/DGKγ and *Zmat4*/ZMAT4. Studies have shown loss of DGKγ is associated with decreased cell density and increased cell soma size [128]. It is possible that upregulation of DGKγ in our data may be compensating for the increased neuronal packing density and decreased soma size observed in RTT [12, 40, 41, 111, 129]. *Zmat4* localizes within cerebellar cortical and granule cell layer inhibitory interneurons [130]. UniProtKB biological and molecular gene ontology terms for *Zmat4* suggest that it may be involved in DNA/RNA binding [131].

Pathways identified from the transcriptome and proteome data sets relating to neuronal and synaptic transmission, damaged synapses, and abnormal neuronal morphology have been previously described [41, 111]. For example, the “damage of synapse” pathway was identified exclusively in the proteome, in which the proteins PrP^C^ (*Prnp*) and PLAA (*Plaa*) were differentially abundant in this pathway. PrP^C^ plays a role in mediating synaptic transmission and long-term potentiation [132]. PLAA is an ubiquitin-binding protein, in which reduced function has led to a disruption in synaptic structure and vesicle recycling in neurons [133]. Additionally, the autism candidate gene *Rab11fip5* was upregulated in our proteome data and plays a role in recycling endosome protein trafficking and neurotransmitter release [134]. Defects in these proteins could affect how neurotransmitters like BDNF, a well-known MeCP2 target reduced in RTT [41, 134, 135], get internalized and processed.

#### Astrocytes

Astrocyte MeCP2 deficiency may contribute to RTT symptoms [14, 15, 18]. Astrocytic mGluR3, a metabotropic receptor known to mediate calcium signals in hippocampal astrocytes [136], was downregulated in our data sets. Sphingosine 1-phosphate receptor 1 (*S1pr1*/S1P1), similarly related to calcium signaling in astrocytes, was also downregulated. While astrocytic calcium signaling has not been directly investigated within the astrocyte RTT field, astrocyte dysregulated calcium signaling has been implicated in related disorders such as Huntington’s disease [137] and epilepsy [138].

Previous work has implicated aberrant astrocyte microtubule dynamics [139, 140] and decreased astrocyte morphological complexity [72] in RTT. The significant gene-protein hit *Slc9a3r1*/NHERF1 is associated with cellular structure/morphology, localizes in the plasma membrane of astrocytes [141, 142], and can transport/anchor G-protein coupled receptors (GPCRs) and ion channels/exchangers to the plasma membrane [143]. We found known protein-binding partners Moesin, Merlin, and PTEN [103, 142, 144], the astrocytic glutamate transporter GLAST [141], and astrocytic-specific GPCRs mGluR3 and S1P1 are disrupted in the proteomic or transcriptomic data sets. It is therefore possible that aberrant changes and interactions between these proteins and NHERF1 could contribute to the observed astrocytic morphological defects in RTT [72].

Finally, pathway analysis of astrocytes confirmed “reactivation of astrocytes” and “morphology of neuroglia” pathways were disrupted. Unlike the vast majority of brain pathological states, our data sets indicated that rather than upregulation of reactive astrocyte markers, markers associated with reactive gliosis (*Gfap*, Gap43, Vim, HSPB1, and ANXA3) were significantly decreased in the RNA-Seq and proteomics data. This suggests that there is a decrease in astrocytic reactivation in RTT, rather than a typical reactive gliosis as seen in most neurological diseases.

#### Microglia

While none of our 35 significant gene-protein matches were considered microglial-enriched, we did identify 10 DE genes and 4 altered proteins (Additional file 3). *C1qa*, *Tyrobp*, and C1QB (all decreased expression) were identified as differentially expressed in RTT patients and are associated with the C1Q complement cascade [33]. Other genes associated with the C1Q complement cascade were also significantly decreased in our transcriptomic data (*Pdgfra* and *Dcn*, Additional file 1). Genetic manipulation of C1Q leads to aberrant pruning by microglia, and excessive engulfment of presynaptic inputs by microglia has been reported in *Mecp2*-null mice [145]. It is also possible that fewer microglia exist in the RTT brain. This has been suggested by Cronk et al. [146] who found microglia are activated and then lost during RTT disease progression. Because our studies were not carried out during the critical developmental period of synaptic refinement, it is unclear how these changes might contribute to altered synapse numbers reported in the RTT brain [147].

#### Oligodendrocytes and their progenitors

One of the shared pathways disrupted in the transcriptome and proteome, “abnormal morphology of oligodendrocytes”, relates to abnormal oligodendrocytes. Many significant glial pathways per data set also related to abnormal migration and myelination/demyelination of oligodendrocytes. In support of these findings, we found a number of proteins associated with myelination that were differentially abundant in our proteomics data (Additional files 3 and 6). Nguyen and colleagues identified MBP and PLP disruptions in *Mecp2*-deficient oligodendrocyte lineage cells [17]. Yet, MeCP2 rescue experiments in oligodendrocytes only partially restored MBP expression while PLP remained abnormally expressed, suggesting other cell types may have a non-cell-autonomous effect on oligodendrocyte lineage cells in RTT [17].

Furthermore, 3 of the 12 novel significant gene-protein hits identified in our data are oligodendrocyte-enriched: *Me3*/NADP-ME3, *Ugt8a*/UGT8, and *Gfra1*/GDNFRα1, in which the former two hits have been implicated in metabolism [94, 97]. Additionally, *Ugt8a* knockout mice present with tremors and ataxia [148, 149], which are common symptoms reported in RTT [135]. GDNFRα1 is a candidate for Hirschsprung’s disease [150], a congenital disorder resulting in loss of nerve cells within the large intestine, and causes constipation, a common symptom of RTT patients [151, 152]. Additionally, the disease/biological function pathway “Hirschsprung’s disease” was also identified in our transcriptome pathway analyses (Additional file 10).

#### Endothelial cells

In this study, we identified a novel and previously identified RTT endothelial-enriched hits where the gene and protein were altered: *Tfrc*/TfR and *Myo1b*/Myo1b, respectively. TfR is a transferrin receptor localized on the plasma membrane of brain capillary endothelial cells and plays a role in transferrin-bound iron transport across the blood-brain barrier (reviewed in [153]). A mutation in WDR45 has been demonstrated in cases of atypical RTT [154, 155], leading to brain iron accumulation [156]. Additionally, one of the pathways identified in the pathway analysis was “permeability of the blood brain barrier” as well as other blood/vasculature pathways (Additional file 10). A more recent study has found that peripheral blood mononuclear cells have an abnormal cellular morphology as well as altered chemokine and cytokine profiles in RTT patients [125]. Additionally, another group has examined mesenteric vessel resistance in RTT mice and found that decreased levels of nitric oxide contribute to endothelial dysfunction [127].

## Conclusions

Overall, we have provided the first comprehensive transcriptome and proteome data set comparison for any RTT model to date, with the goal of providing a more comprehensive view into the biological dysfunction associated with MeCP2 deficiency. Through the integration of these unbiased multi-omics approaches, hundreds of novel genes, proteins, and pathways were identified. Our data indicate that future studies performed in discrete cell populations may provide additional insight into disease biology. By providing this valuable resource, two important types of information are now available for the RTT community: (1) novel gene/protein hits associated with pathways currently under active investigation and (2) new genes, proteins, and pathways that have not been described in RTT and available for open investigation. The reinforcing nature of a combined transcriptomic and proteomic comparison could potentially shed light into future novel therapeutic targets for RTT patients.

## Additional files

Additional file 1: Complete list of significant, differentially expressed (DE) genes identified in *Mecp2* ^*Jae/y*^ cortex. NCBI gene names with WT and *Mecp2* ^*Jae/y*^ FPKM values, Log_2_ fold change expression, and *q*-value (FDR) are provided for each identified DE gene. File format: Microsoft Excel spreadsheet. (XLS 84 kb)

Additional file 2: List of significant genes (*p* < 0.05) identified as RTT hits along with references. Information on WT and *Mecp2* ^*Jae/y*^ FPKM values, Log_2_ fold change expression, *p*-value and *q*-value (FDR) are provided per respective gene (based on NCBI designation). The directions of expression for the RTT hits observed in previous studies (with references) are also provided. File format: Microsoft Excel spreadsheet. (XLS 34 kb)

Additional file 3: List of significant genes and proteins that are CNS cell type-specific. Each individual tab represents one expression data set, with each containing respective gene/protein name and identity, FPKM or relative peptide abundance, fold change expression, estimated size, *p*-, and/or *q*-value (FDR), and which CNS cell population each gene/protein belongs to. File format: Microsoft Excel spreadsheet. (XLS 213 kb)

Additional file 4: CNS cell type-specific genes from Zhang et al.’s study compared to the Sharma et al. study. Significant DE genes (DEG) identified as cell type-specific from the Zhang et al. study [69] were compared to CNS cell type-enriched proteins identified from the Sharma et al. study [60]. Each tab contains information on one specific CNS cell type. Within each tab, information is broken up by 3 parts: part 1 (highlighted in blue)—gene expression information on genes identified in this study, including WT and *Mecp2* ^*Jae/y*^ FPKM values, Log_2_ fold change expression, and *q*-value (FDR); part 2 (highlighted in light orange)—gene identified as CNS cell type-specific in the Zhang et al. study [69] along with the respective gene’s fold change enrichment in the identified cell type relative to all other CNS cell types; and part 3 (highlighted in purple)—gene/protein identified as CNS cell type-specific in the Sharma et al. study [60] along with the respective gene’s fold change enrichment (Log_2_ fold expression and Log_2_ LFQ intensity) in the identified cell type relative to all other CNS cell types, −Log_10_ *p*-value, standard deviation in LFQ intensity, and UniProt accession identity (major protein IDs). For oligodendrocytic-enriched genes, genes enriched in oligodendrocyte precursor cells (OPC), newly formed oligodendrocytes, and myelinating oligodendrocytes from the Zhang et al. study [69] were combined under the broad category “oligodendrocyte-enriched.” For endothelial-enriched genes, only genes identified as endothelial-enriched genes from the Zhang et al. study [69] are provided since the Sharma et al. study [60] did not examine endothelial cells. File format: Microsoft Excel spreadsheet. (XLS 71 kb)

Additional file 5: Gene size and fold change expression breakdown for CNS cell type-specific DE genes. Significant DE genes with CNS cell type-specific designations were broken down into 4 categories—(1) decreased expression, gene size < 100 kb (blue); (2) decreased expression, gene size > 100 kb (light orange); (3) increased expression, gene size < 100 kb (purple); and (4) increased expression, gene size > 100 kb (red). Within each category, DE genes are organized by their respective CNS cell type-specific distribution. The exact Log_2_ fold change and gene size (kb) values are listed for each DE gene. File format: Microsoft Excel spreadsheet. (XLS 66 kb)

Additional file 6: Complete list of significant, differentially abundant proteins identified in *Mecp2* ^*Jae/y*^ cortex. List of 465 significant proteins/PTMs with UniProt accession identity, number (#) of peptides quantified, WT and *Mecp2* ^*Jae/y*^ relative abundances, fold change expression (represented as *Mecp2* ^*Jae/y*^ relative abundance/WT relative abundance), *p*-value, subcellular location, putative function, and hyperlink are provided. File format: Microsoft Excel spreadsheet. (XLS 302 kb)

Additional file 7: Western blot and mass spectrometry chromatogram validation of the selected, significantly expressed protein hits. A. Western blot quantification of 7 proteins selected for validation. All 7 proteins (*x*-axis) were quantified relative to GAPDH (*y*-axis). Gray bars indicate WT expression (*n* = 4), and turquoise bars indicate *Mecp2* ^*Jae/y*^ expression (*n* = 4–5). Asterisks indicate statistically significant genotype expression differences (*p < 0.05). B. Representative Western blot images for selected proteins. Due to space constraints, only Western blot images for 4 out of the 7 proteins are shown. GAPDH was used as the loading control and is shown for each respective group of blots. For the mGluR3 blot, the arrows denote that 2 bands are detected, where the 100-kDa band represents the expected molecular weight (denoted with “*”). For the MeCP2 blot, the top arrow denotes the expected molecular weight, while the bottom arrow indicates the expected GAPDH molecular weight. C. Representative mass spectrometry chromatograms for selected validated proteins. Due to space constraints, only 4 out of the 7 chromatograms for proteins validated by Western blot analysis are shown. The top row represents chromatograms from WT cortex, while the bottom row represents chromatograms from *Mecp2* ^*Jae/y*^ cortex. Each chromatogram shows the relative intensity of the respective protein’s peptide concentration (*y*-axis, in units of 10^3^) in relation to its elution time (*x*-axis, in units of minutes) from the mass spectrometer for 1 biological replicate (for each genotype, the most representative chromatogram out of the 4 biological replicates was chosen). For the CIRBP chromatograms, arrows denote the peaks corresponding specifically to the CIRBP protein. Chromatograms for the remaining 3 proteins (HSPH1, RNPEP, and QDPR) showed similar and notable decreases in peptide changes in *Mecp2* ^*Jae/y*^ cortex (data not shown). File format: TIFF image. (TIFF 1874 kb)

Additional file 8: List of significant proteins/PTMs (*p* < 0.1) identified as RTT hits along with references. Information on WT and *Mecp2* ^*Jae/y*^ relative abundance values, fold change expression, and *p*-value are provided per respective protein/PTM. RTT hits identified from proteomics-based studies are highlighted in yellow. File format: Microsoft Excel spreadsheet. (XLS 33 kb)

Additional file 9: List of 77 gene-protein matches (gene expression *p* < 0.05, protein expression *p* < 0.1). Information on WT and *Mecp2* ^*Jae/y*^ FPKM/relative abundance values, UniProt accession identifier, fold change expression, *p*-, and *q*-value, CNS cell type-specific identity, and any relevant RTT hit references is provided for each gene-protein match. File format: Microsoft Excel spreadsheet. (XLS 55 kb)

Additional file 10: List of all pathways identified in proteomics and RNA-Seq data sets. Pathways identified in either the proteomics or RNA-Seq data sets are provided (“Diseases and Biological Functions”) along with the −Log_10_ *p*-value (i.e., −Log_10_ *p* > 1.3 is the equivalent of *p* < 0.05) associated with each pathway per respective data set. In addition, the number and names of the genes/proteins identified with each respective pathway are listed. File format: Microsoft Excel spreadsheet. (XLS 179 kb)

## Abbreviations

AUC: Area under the curve
BCA: Bicinchoninic acid
CNS: Central nervous system
DE: Differentially expressed
DIA: Data-independent acquisition
FDR: False discovery rate/ *q*-value
FPKM: Fragments per kilobase of transcript per million fragments mapped
IPA: Ingenuity® Pathway Analysis
LC-MS/MS: Liquid chromatography mass spectrometry
PAcIFIC: Precursor acquisition independent from ion count
poly A+: Polyadenylate positive
PTM(s): Post-translational modification(s)
RNA-Seq: RNA sequencing
RTT: Rett syndrome
WCX: Whole cortex
