# Supplementary figures and images for: RNA sequencing and proteomics approaches reveal novel deficits in the cortex of Mecp2-deficient mice, a model for Rett syndrome
Source: Mol Autism. 2017 Oct 24;8:56. doi: 10.1186/s13229-017-0174-4 (PMC5655833; doi:10.1186/s13229-017-0174-4)

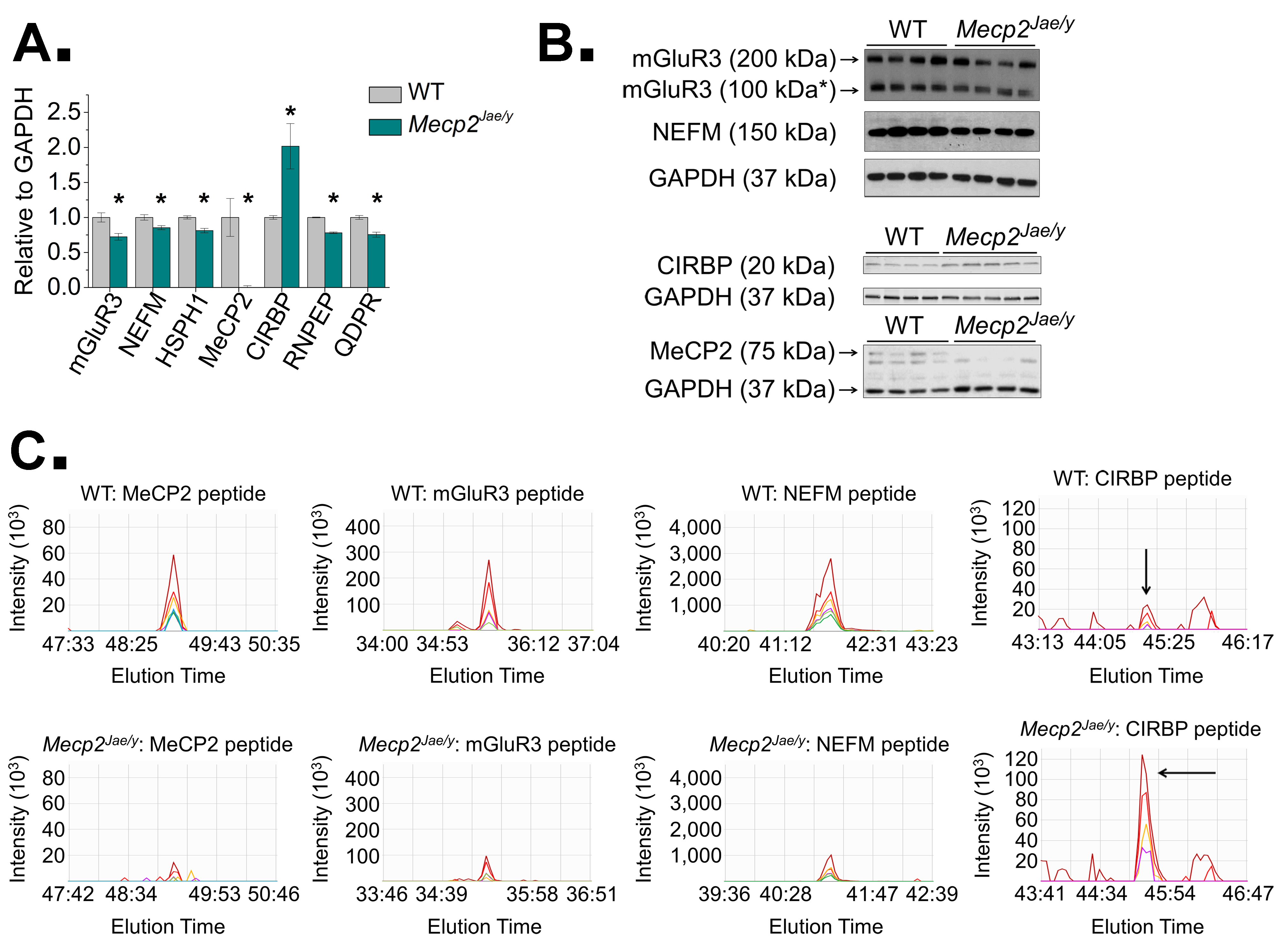

Supplement: Supplementary file 7 — Western blot and mass spectrometry chromatogram validation of the selected, significantly expressed protein hits. A. Western blot quantification of 7 proteins selected for validation. All 7 proteins (x-axis) were quantified relative to GAPDH (y-axis). Gray bars indicate WT expression (n = 4), and turquoise bars indicate Mecp2 Jae/y expression (n = 4–5). Asterisks indicate statistically significant genotype expression differences (*p < 0.05). B. Representative Western blot images for selected proteins. Due to space constraints, only Western blot images for 4 out of the 7 proteins are shown. GAPDH was used as the loading control and is shown for each respective group of blots. For the mGluR3 blot, the arrows denote that 2 bands are detected, where the 100-kDa band represents the expected molecular weight (denoted with “*”). For the MeCP2 blot, the top arrow denotes the expected molecular weight, while the bottom arrow indicates the expected GAPDH molecular weight. C. Representative mass spectrometry chromatograms for selected validated proteins. Due to space constraints, only 4 out of the 7 chromatograms for proteins validated by Western blot analysis are shown. The top row represents chromatograms from WT cortex, while the bottom row represents chromatograms from Mecp2 Jae/y cortex. Each chromatogram shows the relative intensity of the respective protein’s peptide concentration (y-axis, in units of 103) in relation to its elution time (x-axis, in units of minutes) from the mass spectrometer for 1 biological replicate (for each genotype, the most representative chromatogram out of the 4 biological replicates was chosen). For the CIRBP chromatograms, arrows denote the peaks corresponding specifically to the CIRBP protein. Chromatograms for the remaining 3 proteins (HSPH1, RNPEP, and QDPR) showed similar and notable decreases in peptide changes in Mecp2 Jae/y cortex (data not shown). File format: TIFF image. (TIFF 1874 kb) [file 13229_2017_174_MOESM7_ESM.tif]
